# In Vivo Functional Properties of Dairy Bacteria

**DOI:** 10.3390/microorganisms11071787

**Published:** 2023-07-11

**Authors:** Giuseppe Aprea, Ilaria Del Matto, Patrizia Tucci, Lucio Marino, Silvia Scattolini, Franca Rossi

**Affiliations:** Istituto Zooprofilattico Sperimentale dell’Abruzzo e del Molise “G. Caporale”, Campo Boario, 64100 Teramo, Italy; g.aprea@izs.it (G.A.); i.delmatto@izs.it (I.D.M.); p.tucci@izs.it (P.T.); l.marino@izs.it (L.M.); s.scattolini@izs.it (S.S.)

**Keywords:** dairy microorganisms, SLAB, NSLAB, propionibacteria, minor species, in vivo studies

## Abstract

This literature review aimed to collect investigations on the in vivo evidence for bacteria associated with fermented dairy foods to behave as probiotics with beneficial effects in the prevention and treatment of various diseases. All main bacterial groups commonly present in high numbers in fermented milks or cheeses were taken into account, namely starter lactic acid bacteria (SLAB) *Lactobacillus delbrueckii* subsp. *bulgaricus* and *lactis*, *L. helveticus*, *Lactococcus lactis*, *Streptococcus thermophilus*, non-starter LAB (NSLAB) *Lacticaseibacillus* spp., *Lactiplantibacillus plantarum*, dairy propionibacteria, and other less frequently encountered species. Only studies regarding strains of proven dairy origin were considered. Studies in animal models and clinical studies showed that dairy bacteria ameliorate symptoms of inflammatory bowel disease (IBD), mucositis, metabolic syndrome, aging and oxidative stress, cancer, bone diseases, atopic dermatitis, allergies, infections and damage caused by pollutants, mild stress, and depression. Immunomodulation and changes in the intestinal microbiota were the mechanisms most often involved in the observed effects. The results of the studies considered indicated that milk and dairy products are a rich source of beneficial bacteria that should be further exploited to the advantage of human and animal health.

## 1. Introduction

Probiotic foods are currently defined as foods that supply “probiotics” proven to exert health-promoting effects in human trials. These effects must derive at least in part from the microorganisms present and must be distinct from those of the food matrix [1]. For probiotic foods, the recommendation of providing an adequate number of microorganisms per serving, i.e., at least 10^9^ CFU, is re-affirmed, in accordance with the recommended intake for probiotics of the Food and Agricultural Organization of the United Nations (FAO) and the World Health Organization (WHO) [2]. Indeed, the term “probiotic” can be defined as “live microorganisms which when administered in adequate amounts confer a health benefit on the host” (FAO/WHO), later emended by the International Scientific Association for Probiotics and Prebiotics (ISAPP) as “live microorganisms that, when administered in adequate amounts, confer a health benefit on the host” [3]. 

Probiotic benefits include regulation of intestinal transit, normalization of perturbed intestinal microbiota, competitive exclusion of pathogens, hypolipidemic, anti-atherogenic, and antioxidant effects, immunomodulation, and production of bioactive compounds. These effects are strain-specific so they differ for microorganisms belonging to the same species [3].

In vitro assessment of acid and bile salt tolerance prior to undertaking in vivo trials is required to indicate if a potential probiotic is able to survive in the host’s gastrointestinal tract (GIT). In vivo trials in animal models are necessary to predict beneficial effects in human beings and explain those effects by the observation of changes in tissue, cell structure, and biomarker expression in different organs. 

Bacteria naturally present and involved in fermentation and ripening of dairy products comprise strains able to behave as probiotics with efficacy proven in animal models and in clinical trials [4]. For some of these, the ability to produce bacteriocins, such as different nisin forms, lacticin 3147, pediocins, and enterocins, enhanced the beneficial effects against infections and immunomodulation in animal models [5]. Therefore, in this review, recent studies on the health promoting effects exerted in vivo by dairy products containing potential probiotics and by bacteria isolated from dairy products were surveyed to highlight the multiple health benefits deriving from the uptake of bacteria associated to the fermentation and ripening of dairy foods. 

The in vivo studies summarized in this review were retrieved by a Google Scholar (https://scholar.google.com/, accessed on 10 April 2023) and Scopus (https://www.scopus.com/search/form.uri?display=basic#basic, accessed on 15 April 2023) literature database search with the word association “name of bacterial genus”, “in vivo”, and “dairy” or “milk”, “cheese”, “yogurt”, “kefir”, or “fermented milk”. The origin of strains used in the trials, when not specified in the text, was derived by a search in NCBI nucleotide sequence database (https://www.ncbi.nlm.nih.gov/nuccore/, accessed on 27 April 2023) and only articles for which the strain origin could be retrieved were included in the study. The database search was restricted to the years 2019–2023.

All the probiotic microorganisms described in the studies under consideration were preliminarily tested for tolerance to acidic pH and at least 0.3% (*w*/*v*) bile salts, adhesion to human intestinal cell lines, presence of virulence traits, and antibiotic resistance (AR).

Then specific aspects of the probiotic activity were examined, such as pathogen inhibition in vitro or co-aggregation with pathogens, exopolysaccharide (EPS) production, that is involved in immunostimulation and cholesterol assimilation, and bile salts hydrolysis in vitro as from the study of Darwish et al. [6]. Genome sequences are available for many of the strains used in the trials, in accordance with the requirements for use of microorganisms as probiotics and in food products [7].

A recent review by Illikoud et al. [4] summarized the effects exerted in the amelioration of different medical conditions in animal models and clinical studies published until 2021 by bacterial strains belonging to the species used as starters in dairy products, namely *Lactobacillus delbrueckii* subsp. *bulgaricus* and *lactis*, *L. helveticus*, *Lactococcus lactis*, *Propionibacterium freudenreichii*, and *Streptococcus thermophilus*. Results of the in vivo trials summarized in that review article are briefly recalled here. In addition, more recent studies or relevant investigations not reported there are synthetized for the above species. Moreover, this review takes into account the non-starter dairy lactic acid bacteria (NSLAB) represented by the *Lacticaseibacillus* species, *Lactiplantibacillus plantarum*, *Pediococcus acidilactici*, and other species less frequently isolated from dairy products. Some studies on enterococci are also reviewed, though the safety of these microorganisms is controversial. All these bacteria are naturally present in dairy products and possibly ingested in high numbers, close to 10^9^ CFU per serving, likely to influence host health if endowed with functional traits. Preclinical studies in animal models, clinical studies, and applications in the veterinary sector are reported for each bacterial group.

## 2. Fermented Dairy Products

Dairy products were shown to represent a source of bacteria able to establish, at least temporarily, in the human gut. It was found that traditional Pasta Filata fresh cheeses obtained with natural bacterial cultures contributed bacterial components to the intestinal microbiota since identical sequences of selected genetic markers, *rpo*D and *clp*P, discriminant at intra-species level, were found both in the cheese and in healthy children who consumed the cheese for one week. Some of those fecal isolates were obtained after two weeks of cheese administration suspension, showing a good colonization ability [8]. 

An in vivo pilot study involving healthy individuals *Bifidobacterium mongoliense* BMONG18 detected along the whole Parmesan cheese production chain was detected in the feces of all individuals during cheese consumption. After one week of suspension, *B. mongoliense* BMONG18 decreased revealing low persistence capacity in the human gut. Despite the in vivo function of *B. mongoliense* BMONG18 not being elucidated, the study demonstrated that cheese consumption contributes intestinal microbiota components [9]. 

Illikoud et al. [4] inferred from clinical studies regarding the effects on health of fermented dairy products that their deprivation from human diet caused a lower efficiency of the innate immune response and that daily consumption of yogurt containing a conventional starter constituted by *S. thermophilus* and *L. delbrueckii*, counteracted this effect [4]. Six of those studies showed that yogurt, cheese, or sour cream consumption decreased biomarkers of inflammation in different categories of subjects, including obese women with non-alcoholic fatty liver disease and metabolic syndrome, premenopausal women, obese people, normal weight, and overweight volunteers. They also cited a systematic review of the literature considering 10 trials with contrasting conclusions suggesting that the effectiveness of different fermented dairy products can vary, possibly for the different functional activities of the microorganisms present [4]. More recent studies are summarized below.

### 2.1. Amelioration of Intestinal Health

Antimicrobial peptides of the regenerating family member 3 (REG3) family, that maintain the intestinal barrier, are reduced in the small intestines by aging, but the long-term ingestion of yogurt fermented with *L. delbrueckii* subsp. *bulgaricus* 2038 and *S. thermophilus* 1131 by aged mice increased its expression. The REG3 family is induced in the intestinal epithelial cells by interleukin IL-22, predominantly produced by type 3 innate lymphoid cell (ILC3) that is, in turn, induced by IL-23, produced by dendritic cells (DCs) and macrophages after stimulation of Toll-like receptors (TLRs) by bacterial cell component. In the study by Kobayashi et al. [10], oral administration of these strains to specific pathogen-free (SPF) male BALB/c mice led to *Reg3g* induction in the small intestine cells and production of IL-22 and IL-23 with a higher effect exerted by *S. thermophilus* 1131. 

### 2.2. Amelioration of Atopic Dermatitis

Numerous studies have reported beneficial effects of probiotics on atopic dermatitis, e.g., for the widely used probiotic *L. rhamnosus* GG [11]. Based on this evidence, a study aimed to evaluate the effect of cream cheese containing *Lactococcus chungangensis* CAU 28 (CAU 28) (1.4 g/kg/mouse daily) and the dry cells (10^10^ CFU/mouse, daily) of the same strain compared to an untreated positive control and treatment with bepotastine besilate (BB) on the amelioration of atopic dermatitis in female BALB/c mice sensitized with ovalbumin (OVA) when administered for 8 weeks [12].

In that study, it was observed that cytokines produced by regulatory T (Treg) cells, such as IL-10 and IL-1β and Th2 cytokines IL-4 and IL-5 were significantly lower and levels of the Th1 cytokines, IL-12, IFN-γ, and TNF-α were significantly higher in the CAU 28 and CAU 28 cream cheese groups, indicating an enhancement of the Treg-mediated suppression of Th2 immune response [12]. 

The activation of CD 86 T cell protein expression was significantly lower in the CAU 28 cream cheese group and the expression of CD 274 suppressor of adaptive immune response was significantly higher in the CAU 28 and CAU 28 cream cheese groups. Serum IgE levels and eosinophil, neutrophil, lymphocyte, and monocyte percentages significantly decreased in the latter groups. Mast cell accumulation at the dorsal skin and ileal lesions was suppressed in mice treated with CAU 28 and CAU 28 cream cheese and reduced eosinophil infiltration was observed in the dorsal skin lesions of mice treated with CAU 28, CAU 28 cream cheese, and BB [12]. 

High-throughput sequencing of 16S rRNA gene from feces showed a positive correlation between CD 274 and *Bacteroidales*, *Deferribacteraceae*, *Prevotellaceae*, *Oscillospiraceae*, *Rikenellaceae*, and *Veillonellaceae*, while CD 86 levels were correlated with several bacterial families, including *Verrucomicrobiales* and negatively correlated with *Desulfovibrionaceae*. *Bacteroides* and *Akkermansia* were present at significantly higher abundance in the CAU 28 cream cheese-treated group. The levels of short chain fatty acids (SCFA), associated with the maintenance of gut health and positively correlated with *Lactobacillus*, *Bacteroides*, *Ruminococcus*, and *Akkermansia* were found to be higher in the feces of mice treated with CAU 28 cream cheese [12]. SCFAs, mainly acetic, propionic, butyric, and valeric acids, are produced from complex dietary carbohydrates by components of the colon microbiota. These represent an energy supply and exert regulatory and immunomodulatory functions in the intestinal epithelial cells (IEC). Butyrate, produced from acetic acid, lactate, and amino acids, is the preferred energy source for IEC and its metabolism maintains hypoxia-favoring anaerobic commensal bacteria. Butyrate also promotes the differentiation and inhibits the proliferation of intestinal cells in physiological conditions, thus also repressing cancerous cells [13].

### 2.3. Allergy Amelioration

Milk fermentation was found to alleviate indicators of the immune reaction in CMPA. This immune hypersensitivity condition is a complex disorder in which the main symptoms are atopic dermatitis, eczema, asthma, vomiting, and recurrent diarrhea. In CMPA, B cells induced by antigen activated CD4^+^ T helper (Th) cells produce IgE that bind to a high-affinity Fc receptor on the surface of mast cells or basophils that binds to allergen epitopes and triggers the release of inflammatory mediators such as histamine and mast cell protease-1 (MCPT-1). It was shown that fermentation with combined LAB could reduce the antigenicity of cow milk protein by destroying the linear epitopes in vitro [14]. Therefore, *L. helveticus* KLDS 1.8701, selected for antioxidant capacity and isolated from traditional fermented dairy products in Sinkiang, China and endowed with genes coding two cell envelope proteases (CEP), and *L. plantarum* KLDS 1.0386, isolated from an Inner Mongolia traditional fermented dairy product, endowed with 21 peptidase genes and transport systems, were used by Zhao et al. [14] in single or mixed culture for the degradation of α-casein, α-lactoalbumin, and β-lactoglobulin in reconstituted skim milk. The antigenicity of the fermented milks was compared in specific-pathogen-free female Balb/c mice. On days 0, 7, 14, and 21, the mice were sensitized with a mixture of fermented milk containing *L. helveticus* KLDS 1.8701 and *L. plantarum* KLDS 1.0386, alone or in combination, and 10 µg of cholera toxin (CT) as a Th2-polarizing adjuvant. Anaphylactic shock and diarrhea were less severe and the spleen immune indices were lower in the fermented milk groups. Histamine and MCPT-1 were significantly lower in the fermented milk groups, lungs showed a normal alveolar structure without infiltrating inflammatory cells, and the structure of jejunum villi did not present remarkable edema and inflammatory cell infiltration [14]. 

### 2.4. Amelioration of Metabolic Syndrome

The study by Makwana et al. [15] reported that probiotic fermented milk alone (T1) or enriched with 2% whey proteins and soy proteins (T2) and containing 10^8^–10^9^ CFU/mL of lactobacilli could effectively control obesity when administered for 4 weeks to adult male Wistar rats fed with high fat diets (HFD). The fermented milks were prepared with *L. helveticus* MTCC V3, *S. thermophilus* MD2, and *L. rhamnosus* MTCC NS6 isolated from milk and Indian style yogurts. 

In the fermented milk group, weight gain was 46–55% while it increased by 71% in the group receiving only the HFD. The liver weight and the weight of abdominal fat in treatments T1 and T2 were considerably reduced. Treatment with T1 and T2 produced a significant decline in triglycerides (TG) and total cholesterol but did not influence the levels of low-density lipoprotein (LDL). Among alkaline phosphatase (ALP), aspartate aminotransferase (AST), and alanine aminotransferase (ALT) indicators of liver disease, associated more frequently with obese individuals than the normal population, ALT and ALP significantly decreased in T1 and T2. Release of adipocyte leptin, a regulator of food intake and energy utilization associated with hyperleptinemia, was delayed in T1 and T2. Liver sections stained with eosin and hematoxylin for T1 showed fewer and smaller lipid vacuoles. Viable counts of lactobacilli and *S. thermophilus* in feces during the feeding phase were of the order of 7 Log CFU/mL and increased progressively during the feeding period, indicating that the probiotic lactobacilli stably colonized the intestinal tract [15].

### 2.5. Antihypertensive Effects

The benefits of milk fermented by dairy LAB are exerted also by their ability to form substances with the potential to ameliorate disease conditions [16]. An example is given by angiotensin converting enzyme (ACE) inhibitors (ACE-I) that exert antihypertensive effects and the best studied ACE-I-containing foods are fermented dairy products. The formation of these compounds depends on the proteolytic and peptidolytic activity of LAB that generates small peptides from milk proteins, mainly α, β, and κ-caseins, during fermentation. However, the proteolytic systems in different LAB vary not only between species but also between different strains of the same species. Consequently, starter strains producing fermented milk with higher ACE-I properties must be appropriately selected [17]. 

A spontaneously hypertensive rat (SHR) model was used by Glazunova et al. [17] to evaluate the effect on blood pressure of reconstituted skim milk (RSM) fermented by 8 dairy starter LAB (SLAB) strains or non-fermented negative control for four weeks. Rats fed with RSM fermented with *Lactobacillus delbrueckii* Lb100 isolated from commercial yogurt and *Lactococcus lactis* AM1 isolated from amasi showed a significant decrease of systolic pressure (Psyst) down to 154 mmHg at the end of experiment with a ΔPsyst of approximately −17 mmHg. In addition, the *L. delbrueckii* Lb100 group showed also significantly lower levels of total cholesterol and high-density lipoprotein cholesterol (HDL) [17]. 

The antihypertensive effects of fermented milks were reviewed by Beltrán-Barrientos et al. [18], who reported that, beyond ACE inhibitory activity, these effects may also depend on the presence of compounds with antioxidant, nitric oxide production enhancing, and opioid receptor binding activities and on the presence of γ-aminobutyric acid that binds to GABAB receptors. Moreover, antihypertensive effects can derive from compositional changes in gut microbiota. Among naturally fermented milks, kefir was examined in different studies and it was reported that kefir grains administered to SHRs for 60 days determined a significant decrease in blood pressure and an improvement in endothelial dysfunction for a partially restored imbalance between reactive oxygen species and nitric oxide. Moreover, blood pressure was reduced in SHR after nine weeks of treatment with kefir for a reduced cardiac hypertrophy, improved cardiac contractility and regulation of calcium proteins, and signals from the cardio-regulatory regions of the central nervous system (CNS) [18].

In addition, kefir treatment at the intestinal level normalized the number of Paneth cells, producing antimicrobial peptides and active in the regulation of the intestinal microbiota [19], and decreased thickening of the tunica muscularis, thus reducing alterations of the intestinal barrier. Levels of the lipopolysaccharide (LPS) endotoxin, involved in the pathogenesis of hypertension for its ability to activate the Toll-like receptor 4 (TLR4) with production of proinflammatory cytokines, chemokines, and ROS in CNS [20], decreased in the kefir-treated SHR rats. Kefir assumption normalized the levels of inflammatory cytokines TNF-α and IL-6 and reduced neuroinflammation with consequent mitigation of hypertension [18].

In the following sections, in vivo studies regarding single species or genera of dairy bacteria are summarized.

## 3. *Lacticaseibacillus* Species

The *Lacticaseibacillus* genus comprises different species able to exert probiotic functions with a variable asset of genes that confer probiotic activities [21]. *L. paracasei* is the species of non-starter LAB (NSLAB) most frequently isolated from cheeses of different types [7,22,23,24]. 

### 3.1. Improvement of Intestinal Health

Adhesion to the intestine represents a critical parameter for probiotic action. The adhesion ability of *L. casei* ATCC 393 to the gastrointestinal tract of Wistar rats was examined by Saxami et al. [25]. After daily administration for 7 days, strain-specific multiplex PCR on isolates showed that the level of adhesion in cecum and colon was equal to or higher than 6 Log CFU/g, reaching a minimum level required for a probiotic effect. However, the adhesion in GIT was transient, so daily consumption of the specific strain appeared necessary [25]. 

*L. paracasei* CIDCA 8339 with probiotic potential and safe for consumption isolates from kefir grains, when administered in fermented milk at 10^10^ CFU daily to 7 week-old male BALB/c mice in which acute gastric damage was induced with ethanol and HCl, protected gastric mucosa with a significant reduction in the gastric damage in 50% mice [26].

### 3.2. Anti-Cancer Effects

The remarkable anti-tumor, immunomodulatory, and modulation of intestinal microbiota activities of lactobacilli administration has been linked to the increase in stimulatory cytokines that favor cytotoxicity. Consumption of probiotic bacteria has also been linked to the prolonged survival of tumor-bearing mice associated with increased IL-12 production. The combination of increased IL-12 secretion with interferon gamma (IFN-γ) production and augmentation of the natural killer (NK) cells and CD4+ T cells populations has been verified by multiple studies [27]. 

Administration of lactobacilli has been shown to stimulate the production of cytotoxic cells, maturation of Th1 helper T cells, and their cytokine production with activation signaling pathways inducing the differentiation, development, and targeted intra-tumor migration of immune cell populations CD8+ T and NK that increased cancer cell apoptosis through cell interactions and secretion of anti-cancer factors [27].

The protective effect of *L. casei* ATCC 393 against CT26-induced colon carcinoma was evaluated by Aindelis et al. [27] by administering 10^9^ CFU of the strain daily to female BALB/c mice for 13 days. On day 10, 5 × 10^6^ CT26 cells were injected subcutaneously. In *L. casei*-treated mice, a statistically significant increase in IFN-γ was observed in Peyer’s patches 3 and 7 days post inoculation of CT26, and IL-12 levels were found to be elevated on day 13. In spleen cells, a 2.5% increase of CD45+CD8+ T cells and increased IFN-γ production was observed. Levels of IL-12p40 were slightly but significantly increased in tumors and a two-fold increase in Granzyme B accumulation was observed. This protein is considered the main effector of target cell killing by NK cells. It is a serine protease produced also by cytotoxic T lymphocytes that is secreted and translocated through cytotoxic granules into the cytoplasm of tumor cells or cells infected with intracellular pathogens and, by activating or inactivating different proteins, can lead to apoptosis. Therefore, its increase enhances tumor cell killing capacity [28]. Moreover, a four-fold increase in tumor-infiltrating lymphocytes (TILs) CD3^+^CD8^+^ cytotoxic T cells occurred that plays a crucial role in cancer immunotherapy. Caspase 3 was significantly active and Poly (ADP-Ribose) Polymerase 1 (PARP1), a typical apoptotic marker, increased. Accumulation of ligands for the C-C chemokine receptor and receptors that act as signals for the migration of CD8^+^ T cells and NK cells showed that *L. casei* ATCC 393 induced strong Th1 immune responses and impaired tumor growth for the production of immunostimulatory cytokines in lymphatic organs and secretion of molecules that induced the migration of cytotoxic T cells in the tumor. 

This probiotic strain was used to synthesize selenium nanoparticles (SeNps). When administered by oral gavage in male BALB/c mice in which colon cancer was induced by subcutaneous injection of CT26 cells, both 10^8^ CFUs per mL of *L. casei* ATCC 393 and the derived SeNps induced a statistically significant inhibition in tumor growth with a tumor volume reduction of 77% and 54%, respectively [29].

### 3.3. Amelioration of Metabolic Syndrome

*L. rhamnosus* Lb102 isolated from raw milk (acc. n. KJ679486.1) was evaluated in vivo in C57BL/6 mice fed with a high-fat high-sucrose diet for 8 weeks [30]. Mice treated with *L. rhamnosus* Lbl02 strain showed a significant decrease of body weight gain attributable to the reduction of visceral fat, indicating a decreased food efficiency. *L. rhamnosus* Lbl02 improved insulin sensitivity after 6 weeks of administration, proven by a rapid drop of glucose levels at 10 min after insulin injection without any change in fasting glycemia. After 8 weeks of treatment, glucose tolerance improved in an oral glucose tolerance test. *L. rhamnosus* Lbl02 significantly reduced plasma non esterified fatty acid (NEFA) levels. In fecal samples of *L. rhamnosus* Lbl02-supplemented mice, the abundance of genera *Adlercreutzia* and *Clostridium* after 4 weeks and *Streptococcus* and *Lactobacillus* after 8 weeks decreased, while *Anaerovorax* genus was more represented. In gut, treatment with *L. rhamnosus* Lbl02 significantly increased gene expression of zonula occludens 1 (*zo-1*) and occludin, which code for important tight-junction proteins controlling epithelial integrity. Moreover, mucins 2 and 3 (*muc2* and *muc3*) gene expression increased, suggesting that the gut barrier was reinforced by a thicker mucus layer. These results indicated that treatment with *L. rhamnosus* Lbl02 protected gut integrity in an obesogenic diet [30].

### 3.4. Immunostimulation

*L. paracasei* K5 dairy isolate induced a rapid and high level recruitment of leukocytes in the exudates when injected subcutaneously in air pouches in ΒALB/c male mice 3 h post injection, significantly more than *L. casei* ATCC 393 and *L. rhamnosus* GG, with higher levels of granulocyte-colony stimulating factor (G-CSF); interleukins IL-1α, IL-1β, IL-1ra, IL- 6, and IL-16; and chemokines CCL3, CCL4, CXCL1, and CXCL2. Moreover, the administration of *L. paracasei* K5 decreased the expression levels of sICAM, TIMP-1, complement component 5α (C5α), macrophage colony-stimulating factor (M-CSF), and triggered receptor expressed on myeloid cells 1 (TREM-1) [31]. 

Kefir milk prepared from Tibetan Kefir grains containing 26 LAB and 14 yeast strains with or without addition of about 5 × 10^7^ CFU *L. paracasei* Ž2, isolated from the same source, were administered daily to female BALB/c mice for 11 days. The *L. paracasei* group showed the highest level of the helper T lymphocytes (CD4^+^), with increased ratio of CD4^+^:CD8^+^ lymphocyte, indicative of immunostimulation. Mucin MUC-1 was upregulated in the jejunum and MUC-2 and IgA were also upregulated in the cecum and ileum. The highest loads of lactobacilli were noted as biofilm on the surface of the mucin layer in the *L. paracasei* group. From the results, authors suggested that the *L. paracasei* Ž2 strain alone should be used mainly for short-term stimulation of immunocompromised patients [32]. 

### 3.5. Infection Mitigation

As reported by Valente et al. [33], *L. rhamnosus* D1 isolated from Minas cheese showed protection from *L. monocytogenes* infection in four-week-old conventional male BALB/c mice that received the following treatments (whole doses) by gavage each day for 2 weeks: 10^8^ CFU of *L. plantarum* B7 or 10^8^ CFU of *L. rhamnosus* D1, or 100 µL of 0.9% saline (control group). On day 11 of treatment, all animals were infected with 10^6^ CFU of *L. monocytogenes* intravenously. After the challenge, a greater weight loss was observed in the mice in the *L. plantarum* B7 group. In contrast, the *L. rhamnosus* D1 group showed the greatest weight gain, indicating improvements in feed conversion that may help animals to fight infection [33]. 

*L. casei* LC2W, isolated from a traditional Chinese dairy product, showed an inhibitory effect on *E. coli* O157:H7 in vivo. The two strains were labeled with different fluorescent proteins and monitored in the intestinal tracts of live mice using an in vivo imaging system. The results showed that *L. casei* LC2W inhibited the colonization of O157:H7, thus exerting preventive and mitigating effects on the severity of colitis in infected mice [34].

*L. paracasei* Zhang was isolated from koumiss, a traditional drink made from mare’s milk by nomadic populations of China and Mongolia that is believed to exert curative effects on digestive diseases and other diseases, including tuberculosis, bronchitis, and anemia [35]. When 10^10^ CFU of this strain were administered daily for 28 days to young, middle-aged, and elderly individuals, it modulated fecal microbiota by suppressing the potentially harmful *Pseudomonas* and *Acinetobacter* genera, elevating SCFAs for a prolonged period and reducing total bile acids (TBA) [36]. 

*L. paracasei* Zhang alleviated mice mastitis induced by *E. coli* in lactating mice by determining an increased expression of the tight junction proteins claudin-3, occludin, and ZO-1 and decreased expression of the inflammatory cytokines TNF-α, IL-1β, and IL-6 with improvement of the histological score and blood–milk barrier disruption. The treatments compared were *i*, intramammary infusion of 10^5^ CFU *L. paracasei* Zhang alone in one group, *ii*, of 10^3^ CFU *E. coli* alone in another group and *iii*, of 10^5^ CFU *L. paracasei* Zhang and injection of 10^3^ CFU *E. coli* after 24 h in a third group. The latter treatment was aimed at evaluating the effect of *L. paracasei* Zhang against *E. coli* infection by comparison with administration of the probiotic alone or *E. coli* alone. In the control group, only suspension buffer was infused. No redness or swelling was detected in the mammary tissues in the control, *L. paracasei* Zhang, and *L. paracasei* Zhang and *E. coli* groups, while redness and bleeding were observed in the *E. coli* group with remarkable pathological injury and the presence of a large number of neutrophils in the alveolar lumen [37].

### 3.6. Allergy Alleviation

Oral administration of *L. paracasei* Zhang attenuated allergy symptoms and intestinal epithelial damage induced by the allergen tropomyosin (TM) in BALB/c mice, altered the development and function of dendritic cells (DCs), T cells, and B cells, finally resulting in the change of TM-specific antibody isotypes into a tolerogenic pattern [38].

## 4. *Lactiplanibacillus plantarum*

### 4.1. Amelioration of Intestinal Health

Some studies demonstrated the beneficial effects of *L. plantarum* on intestinal health and microbiota composition. Zago et al. [39] found that among 90 strains of *L. plantarum* isolated from different cheeses, three strains with a high degree of agglutination, surface hydrophobicity, and good tolerance to simulated gastric juice showed no microbial translocation to liver and spleen when administered to 6-week-old BALB/c female mice for 2, 5, or 7 days in concentration 10^8^ CFU/mL. The histological examination of the small intestine showed no lymphocyte infiltrates in the villi lamina propria, or the presence of edema or mucosal atrophy. The phagocytic activity of peritoneal macrophages and the number of IgA-producing cells were enhanced for all *L. plantarum* strains and feeding periods but with different profiles of immunostimulation [39]. 

*L. plantarum* C37 supplied at 4 × 10^9^ CFU to Kunming male mice led to an increase of the viable counts of lactobacilli in feces and a decrease of the viable counts of enterococci with no significant effect on the number of bifidobacteria, *Enterobacter*, and *Clostridium perfringens* [40]. 

*L. plantarum* NWAFU-BIO-BS29 isolated from traditional Chinese fermented milk from Gansu province was evaluated for its effect on the intestinal health of mice. It was orally administered daily for two weeks to BALB/c white mice in 10^8^ CFU/day or 8 × 10^9^ CFU/day. The higher dose improved the intestinal length and the average value of the liver and spleen indexes. The *L. plantarum* group showed healthier villi and a normal structure of the intestinal mucosa with no loss of goblet cells. The SCFA concentration in this group, mainly butyric and valeric acids, significantly increased. The *L. plantarum* treatment led to the increased abundance of Firmicutes, Patescibacteria, Campylobacterota, Deferribacterota, Proteobacteria, and Cyanobacteria phyla and genera *Vampirivibrionia*, *Acetivibrio* sp., *Clostridia bacterium*, *rumen bacterium*, *Eubacterium brachy group*, *Halomonas*, *Lactobacillaceae*, *Gastranaerophilales*, *Cyanobacteria*, *Clostridiales bacterium* CIEAF 020, *Lachnoclostridium*, and *Streptococcus*, and decreased the abundance of *Bacteroides* and *Desulfovibrio* [41].

### 4.2. Amelioration of Metabolic Syndrome

Yousef et al. [42] reported that *L. plantarum* YS5, selected on the basis of cholesterol removal ability in vitro, when supplemented in 10^6^–10^7^ CFU/g of feed to male Wistar rats for eight weeks, significantly decreased serum total cholesterol, TG, and LDL levels, and increased HDL to levels higher than observed in mice fed with a normal diet. The rats treated with the high fat diet and supplemented with *L. plantarum* YS5 showed significantly reduced levels of very low-density lipoprotein cholesterol (VLDL) compared with the normal diet group. 

*L. plantarum* 29V isolated from raw milk was added to pasteurized honey, where in 28 days, it declined from 8.49 Log CFU/mL to final numbers of 7.2 and 5.08 Log CFU/g at 4 °C and 25 °C, respectively, and was administered to adult male hypercholesterolemic Wistar rats. During the 28 day treatment, the total cholesterol, (VLDL+LDL)-cholesterol, and TG levels remained lower in treated rats, though with no statistically significant differences. In addition, HDL increased and the atherosclerosis index was significantly lower [43]. 

The application of *L. plantarum* LC38 from camel milk on the wound site in a diabetic rat model significantly enhanced healing activity and accelerated wound closure after 14 days for the capacity to release lactic, succinic, and citric acids [44]. 

In C57BL/6J mice fed a cholesterol-enriched diet, *L. plantarum* Y15, with high bile salt hydrolase (BSH) activity, effectively ameliorated weight gain and alleviated liver histopathological variations. Total cholesterol (TC), TG, and LDL levels were significantly lowered by *L. plantarum* administration. Metagenome analysis based on the V4 region of the 16S rRNA gene of gut microbiota showed that *L. plantarum* Y15 supplementation elevated the diversity of the microbial community and contrasted the increase in Proteobacteria (13.54%), increasing the relative abundances of *Lactobacillus*, *Bifidobacterium*, *Bacteroides*, *Clostridium*, *Prevotella*, and *Oscillospira*. The expression levels of gene encoding FXR and SHP, that increase the catabolism of cholesterol and biosynthesis of bile acids by the α-hydroxylase CYP7A1 in the liver, were reduced by *L. plantarum* Y15 supplementation, while CYP7A1 gene was significantly upregulated, thus indicating that *L. plantarum* Y15 supplementation could lower cholesterol by upregulating cholesterol degradation genes [45].

### 4.3. Anti-Cancer Effects

Oral administration of *L. plantarum* in mice with colon cancer induced by challenge with murine adenocarcinoma CT26 cells led to longer survival of the tumor-bearing mice. Indeed, changes in the tumor microenvironment favored Th1 immunological responses and the recruitment of CD3^+^CD8^+^ cytotoxic T cells and NK cells [46].

### 4.4. Infection Mitigation

In mice infected with *Salmonella* Typhimurium SL1344, the intake of *L. plantarum* strain ACA-DC287 resulted in a decrease in the levels of pathogen cells associated with intestinal tissues and of those present in the intestinal contents [47]. 

In a *Salmonella* Typhimurium challenge, male BALB/c JUnib mice received daily 7 Log CFU of *L. plantarum* B7 from Brazilian artisanal cheese in fermented milk for seven days prior to infection with 5 Log CFU *Salmonella* Typhimurium and for seven days post-infection. Cumulative mortality evolution was delayed and weight loss reduced in mice treated with *L. plantarum* B7. Translocation to liver, which causes the death of mice in this *Salmonella* Typhimurium infection, was also less severe but not translocation to spleen. Increase of ileal levels of IFN-γ and IL-6 at days 5 and 7 after the challenge with *Salmonella* Typhimurium, was prevented possibly for the stimulation of TGF-β gene expression [48]. 

Less inflammatory foci surrounded by necrotic tissue were observed in the liver and less augmented Payer patches and necrosis in mucosa were evident in the ileum. Intestinal villi height was higher in mice treated with *L. plantarum* B7. In the ileum of one animal that received *L. plantarum* B7, a higher diversity among the predominant ribosomal sequence variants (RSVs) and a lower abundance of the *Salmonella* genus RSV was observed, indicating that mechanisms other than direct pathogen reduction are associated with the ameliorated clinical and histopathological parameters [48]. 

*L. plantarum* DR7 strain was administered for 12 weeks in a double-blind RCT clinical study to young and middle-aged adults, and in young adults it marginally reduced the duration for nasal, pharyngeal, and general flu symptoms, while in middle-aged adults, it significantly reduced the duration of nasal symptoms and marginally reduced the duration of general flu symptoms and frequency of upper respiratory tract infection (URTI) in the 12 weeks study period. Plasma anti-inflammatory cytokines such as IL-4 and IL-10 increased in young adults, but not in middle-aged adults. Plasma proinflammatory cytokines IFN-γ, TNF-α, and IL-1β were reduced in middle-aged adults, while only IFN-γ was reduced in young adults. Therefore, in general, *L. plantarum* DR7 alleviated inflammatory parameters [49]. 

The administration of *L. plantarum* DR7 did not affect antioxidant potential in plasma, red blood cell (RBC) membrane, or hemolysate in young adults, marginally increased the antioxidant potential of RBC membranes in middle-aged adults and significantly increased this factor in all subjects. Moreover, it decreased the concentration of malondialdehyde (MDA), an indicator of lipid peroxidation that increases in many diseases [50], in all subjects, upregulated the expression of plasma genes for CD44 and CD117 in young adults, and downregulated the expression of plasma genes for CD4 and CD8. The administration of *L. plantarum* DR7 downregulated the expression of plasma genes for NKp46 and NKp30 in young adults and CD56, NKp46, and NKp30 in middle-aged adults. In general, the administration of *L. plantarum* DR7 led to less activation of T cells, with more non-resting and mature natural killer (NK) cells. Therefore, it was concluded that *L. plantarum* DR7 can protect adult populations against URTI by inducing immunomodulatory and anti-inflammatory effects while promoting mucosal barrier integrity and action of NK cells [49].

### 4.5. Amelioration of Aging Damages

D-galactose is an aging-inducing agent that gives rise to a process similar to natural aging in animal models. Indeed, only a small amount of it is converted into glucose and metabolized, while a large amount remains unaltered causing the formation of large amounts of superoxide anions and oxidation products that damage cell macromolecules [51]. 

Zhang et al. [51] examined the in vivo antioxidant and gut microbiota regulation effects of its component EPS 1from *L. plantarum* YW11 in male mice in which oxidative stress was induced with D-galactose. The mice were subcutaneously injected with D-galactose solution and then received vitamin C (VC), to serve as anti-aging control or low-dose (LD) or high-dose (HD) EPS, or fermented milk (FM) containing 8.41 Log CFU/mL of viable *L. plantarum* YW11 for 12 weeks. The MDA levels of the HD group and fermented milk were similar to those of the VC, indicating an effective blocking of lipid oxidation in serum. The HD group had the highest serum activities of GSH-Px, SOD, CAT, and T-AOC and the CAT activities were higher than those in the VC group. Those enzyme activities were higher in the FM group than in the LD, indicating a dose-dependent antioxidant activity of EPS [51]. 

Zhao et al. [52] used D-galactose induced aging in Kunming mice to test the anti-aging effect of *L. plantarum* KSFY02 isolated from naturally fermented Xinjiang yogurt. Compared treatments were a normal group with no treatment, a positive control supplemented only with D-galactose, a group supplemented daily with 10^9^ CFU/kg of body weight of a *L. delbrueckii* subsp. *bulgaricus* strain, a group supplemented daily with 10^9^ CFU/kg of body weight of *L. plantarum* KSFY02, and a group supplemented with vitamin C as anti-aging agent. After four weeks, the mice were intraperitoneally injected with D-galactose for 6 weeks, while the other treatments were continued. At the end of treatment, the indices of thymic, cerebral, cardiac, liver, spleen, and kidney indices of atrophy mice in the LAB and vitamin C treated groups were significantly higher than those of the control group, particularly in the *L. plantarum* LP-KSFY02 group. In this group, contents of nitric oxide (NO), MDA, GSH, and activities of the superoxide dismutase (SOD) and glutathione peroxidase (GSH-Px) in the serum were the most similar to the normal group. Mice in the anti-aging treatment groups had fewer lipid vacuoles, indicating alleviated endosmosis, defined boundary between the epidermis and dermis, alleviated cell swelling, and inflammatory infiltration, in particular in the *L. plantarum* LP-KSFY02 group. Spleen lesions were found to be lowest in the *L. plantarum* LP-KSFY02 treatment. The mRNA expression levels for *Nos1* (neuronal nitric oxide synthase), *Nos3* (endothelial nitric oxide synthase), *Sod1* (cuprozinc-superoxide dismutase), *Sod2* (manganese superoxide dismutase), *Cat* (catalase), *Hmox1* (heme oxygenase-1), *Nfe2l2* (nuclear factor-erythroid 2 related factor 2), *Gclm* (γ-glutamylcysteine synthetase), and *Nqo1* (NAD(P)H dehydrogenase [quinone]1) encoding proteins with anti-oxidative activities in liver and spleen increased significantly in all the anti-aging treatment groups. Expression of SOD 1 and 2, catalase (CAT), GSH 1 and 2, and β-actin (ACTB) in liver and spleen was stronger in the *L. plantarum* LP-KSFY02 group. Therefore, it could be concluded that the efficacy of *L. plantarum* LP-KSFY02 was significantly higher than the other treatments in reverting the damages of D-galactose induced aging [52].

### 4.6. Improvement of Stress Symptoms

Liu et al. [53] carried out the first clinical study to correlate the effects of a probiotic towards gut microbiota changes and brain neurotransmitters genes, strengthening the hypothesis of health benefits along the gut–brain axis. *L. plantarum* DR7 isolated from fresh cow’s milk was evaluated in a double-blind, RCT design study randomized on men or women aged 18–60 years old and who scored moderate stress level on Cohen’s Perceived Stress Scale (PSS-10) [53]. 

Subjects with chronic diseases, medication assumption, and deficient in glucose-6-phosphate dehydrogenase were excluded. Daily consumption consisted of either 10^9^ CFU/sachet of DR7 powder with maltodextrin excipient or placebo with only maltodextrin. Based on a gastrointestinal questionnaire, *L. plantarum* DR7 decreased the frequency of defecation in all subjects, possibly by modulating the increased bowel movement triggered by the central nervous system (CNS) upon stress. No significant effects against pain and discomfort were registered in normal adults aged above 30 years old, but younger adults below 30 years reported decreased durations for both direct and indirect gastrointestinal symptoms from week-4. After *L. plantarum* DR7 administration, significant differences were consistently observed in the fecal microbiota, such as reduced abundance of Clostridiales, larger decrease of Selenomonadales, that exert detrimental effects on the host, increased abundance of Deltaproteobacteria and with genuses Bilophila and *Desulfovibrio*, producing H_2_S that is an important mediator for epithelial injury repair, dysbiosis prevention, and inflammation resolution, and decrease of *Actinomyces*. The latter genus is able to cause actinomycosis due to its ability to form biofilm and induce and aggravate inflammation. Neurotransmitters β-hydroxylase (DBH), indoleamine 2,3-dioxygenase (TPH2), and tryptophan 2,3-dioxygenase (TDO) involved in the serotonin and dopamine pathways were differently regulated and changes correlated to changes in the relative abundance of specific bacterial groups in the gut microbiota. *Bacteroides* and Desulfovibrionales, which were higher in the *L. plantarum* DR7 group, were negatively correlated with DBH, which catalyzes the conversion of dopamine to norepinephrine, an indication of increased stress, and positively correlated with TPH2, which converts tryptophan to serotonin in the brain, where an imbalanced level of serotonin has been reported in patients with psychological disorders including mood perturbation and anxiety. DBH was positively correlated with Clostridia [53]. 

Anxiety is the first psychological reaction to stress and, if prolonged, can lead to mental illnesses including depression, which affects over hundreds of millions of people globally. Stress responses activate the autonomic nervous system and the hypothalamic–pituitary–adrenal axis with increased levels of catecholamines and glucocorticoids in blood and tissues. These hormones alter immune functions, such as antigen presentation, leukocyte trafficking and proliferation, antibody secretion, and cytokine release. Long-term exposure to glucocorticoids leads to increased resistance of the glucocorticoid receptors with consequent decrease of immune cells sensitivity and activation of inflammatory responses. *L. plantarum* DR7 activated the 5′-AMP-activated protein kinase (AMPK) pathway via phosphorylation. AMPK is inactivated in mice by chronic mild stress, inducing anxiety and depression-like behaviors [54]. 

Therefore, Chong et al. [54] evaluated the effects of *L. plantarum* DR7 on stress, anxiety, depression, memory capacity, and cognitive functions in stressed adults in a double-blind, randomized, and placebo-controlled design study. The study participants and probiotic and placebo administration were as in the study described above. 

The perceived stress score did not vary significantly in the two groups in 12 weeks. Instead, scores from the Depression, Anxiety, and Stress Scale (DASS-42) questionnaires indicated that the administration of *L. plantarum* DR7 reduced scores in all age groups after 8 weeks. Administration of *L. plantarum* DR7 significantly reduced plasma cortisol, which activates the brain noradrenergic system triggering alertness, awareness, wakefulness, and attention, and reduced pro-inflammatory cytokines levels in all subjects and increased the anti-inflammatory cytokine IL-10. The latter increased mostly in young adults after 12 weeks, while for the other subjects only a decrease of pro-inflammatory cytokines IFN-γ, TNF-α, and IL-1β was observed [54]. 

The administration of *L. plantarum* DR7 enhanced social emotional cognition, verbal learning, and memory, and marginally reduced errors for associate learning in all subjects over 12 weeks, benefiting mostly older adults [54]. 

The administration of *L. plantarum* DR7 lowered the expressions of plasma neurotransmitters dopamine β-hydroxylase (DBH) and tryptophan 2,3-dioxygenase (TDO) and increased the expression of tryptophan hydroxylase-2 (TPH2) and 5-hydroxytryptamine receptor-6 (5-HT6) over 12 weeks in all subjects. These data indicated that higher expressions of TPH and 5-HT6 potentially channeled tryptophan towards the production of serotonin [54]. The latter can exert anxiety inhibiting effects [55].

## 5. *Lactobacillus delbrueckii* subsp. *bulgaricus* and *lactis*

The subspecies *bulgaricus* and *lactis* of *L. delbrueckii* are commonly associated with dairy products. As reported by Illikoud et al. [4], *L. delbrueckii* subsp. *bulgaricus* was shown to inhibit the development of colitis-associated cancer induced in mice with azoxymethane and DSS by reducing tumor volume, attenuating the intestinal inflammation, with decreased levels of pro-inflammatory cytokines IL-6, TNF-α, IL-17, IL-23, and IL-1β. Strains of *L. delbrueckii* subsp. *bulgaricus* restored antioxidant enzyme activities in induced colitis in rats and, in combination with *L. fermentum* for eight weeks, favored healing in ulcerative colitis patients by decreasing the expression of IL-6, TNF-α, and NF-κ*B* p65 and limiting leukocyte recruitment in the colon and the level of fecal calprotectin. In the elderly, oral supplementation of *L. delbrueckii* subsp. *bulgaricus* enhanced systemic immunity with increased NK cells and antimicrobial peptide hBD-2 and decreased IL-8 in blood, increased the concentration of saliva IgA, and natural killer (NK) cell activity and enhanced the quality of life (QOL) score. Strains of *L. delbrueckii* subsp. *lactis* were shown to mitigate gut inflammation in mice by reducing the macroscopic and microscopic symptoms of DSS-induced colitis, diminishing weight loss, improving survival, and modulating the production of cytokines TGF-β, IL-6, and IL-12 in the colon, and TGF-β and IL-6 in the spleen with expansion of CD4^+^FOXP3^+^ regulatory T cells in the cecal lymphnodes [4].

### Alleviation of Hepatic Injury

The strain *L. delbrueckii* subsp. *bulgaricus* L7, isolated from traditional fermented Xinjiang cheese, showed relevant antioxidant capacity based on 2,2-Diphenyl-1-picrylhydrazyl (DPPH) radical scavenging activity, reduction ability, trolox equivalent antioxidant capacity (TEAC), and antioxidant capacity in milk, so Liu et al. [56] evaluated its effects in alcohol-induced oxidative stress and inflammatory responses in acute alcoholic liver disease (ALD) in C57BL/6 male mice [56]. 

The mice group fed with *L. delbrueckii* subsp. *bulgaricus* L7 fermented milk presented remarkably decreased oxidative stress in liver tissues caused by alcohol intake showing levels of MDA, SOD, and GSH-Px activities comparable with the normal group and a group supplemented with glutathione (GSH). Serum levels of the SCFAs butyric and acetic acids, significantly reduced by alcohol feeding, were restored at normal level by the fermented milk and GSH uptake. SCFAs production is critical in preventing and decreasing inflammation and can suppress the growth of pathogenic intestinal bacteria, modulate gut-microbiota, improve lipid metabolism and the human immune function, lower intestinal pH, and promote the bioavailability of minerals such as magnesium and calcium [56]. 

Metagenomic analysis of the 16S rRNA gene hypervariable region V3–V4 showed that *L. delbrueckii* subsp. *bulgaricus* L7 administration, but not GSH, better contrasted gut dysbiosis with a decreased abundance of potential pathogens *Porphyromonas* sp. and *Enterococcus* spp., decreased Firmicutes/Bacteroidetes ratio, and a less remarkable decrease of the genus *Ligilactobacillus* induced by alcohol. Alcohol feeding reduced the abundance of Comamonadaceae, and enriched Muribaculaceae and Clostridiales. These changes were less evident in mice fed with *L. delbrueckii* subsp. *bulgaricus* L7 and GSH. In conclusion, *L. delbrueckii* subsp. *bulgaricus* L7 exhibited a better capability than GSH to restore alcohol-induced gut dysbiosis with potential protective effects on alcohol-induced hepatic damage [56]. 

## 6. *Lactobacillus helveticus*

Based on the studies summarized by Illikoud et al. [4], dairy strains of *Lactobacillus helveticus* exerted anti-inflammatory properties in TNBS-induced colitis in mice with reduction of histological damage scores and weight loss, colon shortening, bleeding, and diarrhea. In addition, *L. helveticus* strains suppressed tumorigenesis and carcinogenesis in colorectal cancer induced with azoxymethane and DSS in mice by modulating inflammation with reduction of enterocytes and IL-17-producing T cells proliferation, NF-κ*B* activation, enhanced production of IL-10, and improving microbial homeostasis [4]. 

In healthy mice, *L. helveticus* induced Treg, which regulated immune and inflammatory responses, suppressed the production of IL-17, IL-4, and IL-10, and induced the anti-inflammatory cytokine transforming growth factor-β1 in Peyer’s patch cells. Intraperitoneal and oral administration of a *L. helveticus* strain strongly alleviated symptoms of collagen-induced arthritis (CIA) in mice for the ability to decrease the abundance of immune cells and the subsequent production of collagen type II (CII) specific antibodies and IL-6 [4].

In clinical studies, selected strains of *L. helveticus* prevented the anxiety-like behavior and negative effects on memory induced by a Western-style diet and improved behavioral, cognitive, and biochemical aberrations caused by chronic restraint stress in rats correlated with lower plasma corticosterone, adrenocorticotropic hormone (ACTH), and IL-10 levels in plasma, restoring hippocampal serotonin and norepinephrine levels. In RCT trials, *L. helveticus* strains modulated mucosal and humoral immunity by increasing specific IgG and IgA, reduced the duration of respiratory infections, and alleviated perennial allergic rhinitis in adults by suppressing eosinophils [4]. 

### 6.1. Amelioration of Intestinal Health

Ulcerative colitis (UC) is a non-specific chronic inflammatory disease causing colon and rectum lesions, bloody diarrhea, abdominal pain, weight loss, and ulcer. It is commonly treated with 5-aminosalycilates, glucocorticoids, and immunosuppressants, which only alleviate the clinical symptoms and have side effects [57].

With the aim of developing a safer and more efficient UC treatment agent, EPS-1 produced by *L. helveticus* KLDS1.8701 was administered to C57BL/6 mice for 21 days and DSS colitis was induced on day 15. EPS-1 administration could effectively alleviate induced UC by reversing weight loss and colon shortening. After being treated with EPS-1, the colonic tissues showed reduced structural damage and inflammatory cell infiltration. Levels of TNF-α, IL-1β, and IL-6 were lower and those of IL-10 were higher in the colon in the EPS-1 group. The expression levels of ZO-1, Occludin, Claudin-1, and MUC2 were significantly reversed by the EPS-1 administration. EPS-1 administration significantly increased the level of acetate and butyrate SCFAs in colon [57].

Inflammatory bowel disease (IBD) comprises a range of chronic inflammations and disorders of the gastrointestinal tract, whose most common forms are Crohn’s disease (CD) and ulcerative colitis (UC). Anti-inflammatory agents are traditionally used to treat IBD, but alternative therapies, including probiotics, are evaluated [58]. *L. helveticus* ASCC 511 (LH511) was obtained from the Dairy Innovation Australia Limited (ASCC, Werribee, Victoria, Australia). Citrulline is a non-essential amino acid that was found to ameliorate the inflammatory response by reducing the level of pro-inflammatory cytokines TNF-α, IL-6, and IFN-γ in intestinal ischemia/reperfusion model. Citrulline is produced simultaneously by nitric oxide synthase (NOS) during NO formation from arginine, and then arginine is re-generated from citrulline by argininosuccinate synthetase (ASS) and argininosuccinate lyase (ASL). Some lactobacilli are able to utilize arginine by the arginine deiminase (ADI) pathway. The potential enhancement effect of combination of lactobacilli with arginine in vivo has been investigated, and a synergistic effect was shown to increase protection against liver injury induced by D-galactosamine, high cholesterol diet, and endotoxin, as well as to reduce bacterial translocation [58]. 

In male C57BL/6J mice with colitis induction for 5 days with DSS, fermented milk containing *L. helveticus* ASCC 511 (LHFM) or *L. helveticus* ASCC 511 plus citrulline (Cit_LHFM) was supplied for 12 days. All milk treatment groups attenuated the weight loss. In the LHFM and Cit_LHFM, the survival rate increased to 50% and 70% at day 12, respectively. Cit_LHFM improved the colonic permeability as shown by the reduced concentration of the fluorescent drug FITC-dextran administered to the mice in blood. Treatments with Cit_milk, LHFM, and Cit_LHFM reduced the damage in the distal colon mucosa and showed the lowest score. LHFM and Cit_LHFM treatments decreased disease activity indexes, limited colon shortening, and reduced the ration between colon weight and length (CW:CL). Treatment Cit_LHFM suppressed the expression of IL-4 and IL-17 induced by DSS in serum and all treatments reduced the expression of IL-6, TNF-α, and IFN-γ. LHFM upregulated the expression of IL-10. The highest remediation effect on the mRNA levels of all tight junction (TJ) proteins was observed in Cit_LHFM. Therefore, treatment with Cit_LHFM showed synergistic and superior effects on reinforcing intestinal structure and reducing inflammation [58]. 

### 6.2. Immunomodulation

Li et al. aimed to identify the most efficient bifunctional strain among 265 LAB from fermented foods based on immunomodulatory activity and fermentative ability. One selected strain, identified as *L. helveticus* WHH2580, was used for milk fermentation and the fermented milk was administered to specific pathogen-free male BALB/c mice; on days 28 and 29 of the administration period, mice were submitted to immunosuppression with CTX for 2 days. No significant difference was observed in the body weight between groups receiving fermented milk group or non-fermented milk throughout the administration period. In the fermented milk group, a higher splenic lymphocyte proliferation and a stronger splenic NK cell cytotoxicity activity were observed [59]. 

### 6.3. Allergy Alleviation

*L. helveticus* SBT2171 (LH2171) showed an ameliorative effect on the nasal and ocular symptoms of patients with mite and house dust allergy. Therefore, a study was carried out using a previously established murine model for pollen, ovalbumin (OVA)-specific TCR-transgenic DO11.10 female mice whose T cells recognize OVA323-339. Mice inhaled OVA/saline for 1 h, twice a week for six weeks while freeze-dried *L. helveticus* LH2171 was given to the mice such that they could access it freely during the period of pollen allergy induction. It was observed that face scratching and sneezing were significantly reduced by *L. helveticus* LH2171 administration. Since the treatment did not suppress IgE production, a mechanism other than IgE inhibition was implied [60].

House dust or indoor dust is a mixture of mites, ticks, human scraps, molds, and bacteria with mites *Dermatophagoides farinae* and *D. pteronyssinus* as the most common allergen source. Allergic symptoms include runny nose, sneezing, and ocular symptoms, such as itching eyes and eye swelling [61]. 

Yamashita et al. [61] investigated in a randomized, double-blind, placebo-controlled, parallel group comparative study whether drinkable yogurt (DY) containing *L. helveticus* LH2171 improved QOL in the subjects with daily nasal and ocular discomfort and immune markers in the blood. Active DY, containing *L. helveticus* LH2171, was also fermented by a strain of *S. thermophilus*, while only the latter was present in the placebo DY. Total LAB number was about 10^9^ CFU/g and the *L. helveticus* LH2171 number was 10^7^ CFU/g at the end of the consumption period [61]. 

Decreases in total scores of nasal and ocular discomforts at week 8 were significant in the *L. helveticus* LH2171 group and even more on week 12. Outdoor activity and sleep scores were significantly improved in the *L. helveticus* LH2171 group. The number of sneezes also decreased significantly but not total and antigen-specific IgE levels at any experimental point. No serious adverse or side effects were noted by the responsible physician [61].

Allergy caused by house dust affects approximately 40% of the global population in the industrialized world and the major mechanism of the allergic response is the activation of T helper type Th2 cytokine production, which induces the production of antigen-specific immunoglobulin IgEs by B cells. Pharmacotherapy for perennial allergy, includes use of inhibitors of chemical mediators, anti-histaminic drugs, and topical steroids that can have long-term side effects. Monoclonal antibodies anti-human IgE or receptor antagonists that regulate the signaling IL-4 and IL-13 pathways are expensive alternatives used to treat particular conditions. Noticeably, approaches that can completely cure allergies are still lacking [62]. Following the study by Yamashita et al. [61] on the effect of *L. helveticus* LH2171 in house dust allergy, another RCT was conducted to clarify the anti-allergic mechanism and evaluate the effect on the QOL with 16 weeks test period and use of the profile of mood states 2 questionnaire. Patients involved were 20–65 years old males and females who did not have chronic diseases and presented allergic rhinitis symptoms, with no significant differences between groups in baseline symptoms [62].

In the *L. helveticus* LH2171 group, the severity of allergic rhinitis significantly improved in 4–16 weeks, with significantly improved aspect of the nasal mucus, improved aqueous secretion, and stuffy nose scores. The eosinophil score in the nasal fluids was significantly lower at 4 and 12 weeks. There were no significant differences in allergen-specific IgE and total IgE levels in the serum, but the level of thymus and activation-regulated chemokine (TARC) was significantly lower at eight weeks in the *L. helveticus* LH2171. Eye itching significantly improved in the *L. helveticus* LH2171 group and the severity of nasal symptoms decreased in 16.0% subjects in the placebo group and in 36.8% subjects in the *L. helveticus* LH2171 group. It was concluded that the probiotic strain ameliorated nasal discomfort probably by suppressing eosinophils [62].

### 6.4. Infection Mitigation

Defensins are one of the major groups of antimicrobial peptides in mammals and play critical roles in host defense. Β-Defensins, produced in epithelial cells, are widely distributed in the body and strains of lactic acid bacteria have been reported to induce their expression. Since β-defensins are effective against *P. gingivalis*, an oral bacterium that contributes to the development of periodontal disease, increased expression of β-defensins can suppress periodontal disease [63]. Therefore, Kobatake et al. [63] investigated the expression of β-defensins in female BALB/c mice orally administered with 10^9^ CFU daily of *L. helveticus* SBT2171 for 5 weeks. Three weeks later, 10^8^ CFU of *P. gingivalis* strain 381 was inoculated in the oral cavity of the mice daily for 2 weeks. On day 30, alveolar bone loss was significantly suppressed in the *L. helveticus* group and the abundance of *P. gingivalis*-specific 16S rRNA isolated from gingival tissue was reduced. Furthermore, mRNA levels of *Tnf*α in gingival tissue were lower in the *L. helveticus* group on day 1. Therefore, oral administration of *L. helveticus* SBT2171 ameliorated gingival inflammation caused by *P. gingivalis* Pg381 [63].

*L. helveticus* SBT2171 was administered to Sprague Dawley female rats of specific pathogen-free grade in amount of 10^9^ CFU daily for 35 days, and 10^8^ CFU/mL of *Aggregatibacter actinomycetemcomitans* HK1651 periodontitis agents were given orally from the 21st day, daily for 14 days. It was found that *L. helveticus* SBT2171 reduced the distance from the cement–enamel junction (CEJ) to the alveolar bone crest (ABC), indicating improved alveolar bone resorption. In addition, neat arrangement of periodontal fibers, and small blood vessels proliferation in connective tissue were observed. These results showed that *L. helveticus* SBT2171 attenuated damage to the hemimaxillary tissue in periodontitis, determining reduced inflammatory cell infiltration. The number of *A. actinomycetemcomitans* in the *L. helveticus* SBT2171 group was notably decreased, as well as the expression in the gingival tissue of IL-1 β, IL-6, which significantly increases in patients with periodontal diseases, and TNF- α, involved in chronic inflammation that leads to periodontal tissue damage. Moreover, the expression level of β-defensins significantly increased in the gingival tissues. Therefore, *L. helveticus* SBT2171 improved periodontal disease by increasing the expression of β-defensins and reducing the number of *A. actinomycetemcomitans* [64].

Common carp, *Cyprinus carpio*, is widely cultivated in the world but its farm production is challenged by infectious agents such as *Aeromonas hydrophila* and use of antibiotics leads to unwanted side effects such as immunosuppression, increasing of pathogen multi-drug resistance, and health risks for consumers. Gum Arabic (GA) is a natural prebiotic secreted from Acacia senegal and Sengalia senegal trees and is a safe feed additive in food. It contains non-digestible glucuronic acid, rhamnose, and galactose which can be used as an energy source for gut bacteria and can be converted by the gut microbiota in SCFAs [65]. In the study by Yousefi et al. [65], the *L. helveticus* type strain ATCC 15009, isolated from Emmental cheese, was administered in different combinations with GA to common carp juveniles that were fed ad libitum with different diets C (basal diet), LH1 (10^7^ CFU/g), LH2 (10^9^ CFU/g), GA1 (0.5% *w*/*w*), GA2 (1% *w*/*w*), LH1+GA1, and LH2+GA2, three times daily. After two weeks, the fish were transferred to challenge tanks and exposed to *A. hydrophila* via intraperitoneal injection. Final weight and weight gain rate were significantly higher in the fish fed diets containing prebiotics GA1 and GA2, and synbiotics GA1+LH1 and GA2+LH2 than the control group and the highest value was obtained in the LH1+GA1 treatment. Dietary GA and/or LH supplementation significantly increased the population of LAB in the fish intestine with no significant difference among the treatments. The treatments enhanced white blood cells, with a peak for LH1+GA1. Monocyte percentage was significantly greater in LH1 and synbiotics with also an increase of total serum Ig levels and alternative complement ACH50 activity. Alkaline phosphatase (ALP), lysozyme, and Ig increased in skin mucosa. GA and/or LH diets significantly increased serum SOD and CAT activities and reduced MDA, except for LH2 treatment. The mortality rate of common carp infected with *A. hydrophila* at the end of the infection period showed significant decreases in all experimental groups with lowest levels for GA2. The synbiotic with lower LH and GA improved growth rate and disease resistance as efficiently as higher concentrations in carps [65].

### 6.5. Alleviation of Aging Damages

Strain *L. helveticus* KLDS1.8701 with high antioxidant capacity was administered to mice receiving D-galactose once daily for 8 weeks to induce aging. *L. helveticus* KLDS1.8701 supplementation significantly decreased organic index, liver injury, and endotoxin, and reduced hepatic oxidative stress by modulating the Nrf-2 pathway. *L. helveticus* KLDS1.8701 restored the gut microbiota with increased butyrate production and decreased endotoxin production indicating that attenuation of hepatic oxidative stress was induced by the metabolites of gut microbiota [66].

The EPS purified from the culture of *L. helveticus* KLDS1.8701 exhibited strong scavenging properties on 2,2-diphenyl-1-picrylhydrazyl radical, superoxide radical, hydroxyl radical, and chelating activity on ferrous ion. When supplemented in vivo, the fraction EPS-1 significantly alleviated D-galactose induced liver damage by decreasing organic index, liver injury, and liver oxidative stress. EPS-1 supplementation shifted the gut microbiota composition to that of the control group indicating that the mitigation of hepatic oxidative stress depended on the action on gut microbiota [67].

### 6.6. Improvement of Stress Symptoms

Ingestion of 0.2 mL of 10^9^ CFU/mL of *L. helveticus* WHH1889 isolated from yogurt for five weeks in an induced chronic unpredictable mild stress (CUMS) mouse model of depression reversed the elevated level of serum corticosterone and decreased levels of hippocampal 5-hydroxytryptamine (5-HT) and its precursor 5-HTP. Moreover, the *L. helveticus* treatment significantly improved depressive and anxiety behaviors in mice. Furthermore, the intestinal microbiota diversity, reduced in CUMS mice, was improved by *L. helveticus* WHH1889, with increased colonic 5-HTP level and tryptophan hydroxylase 1 *Tph1* gene expression. The findings indicated that the *L. helveticus* WHH1889 antidepressant-like effects were associated with the modulations of the 5-HT/5-HTP metabolism and gut microbiome. Similar results were obtained with the yogurt isolate *L. lactis* WHH1889, thus showing that different dairy isolates can relieve the symptoms of stress-induced depression [68].

### 6.7. Benefits in Animal Production

The intestinal health of fish is closely related to the immune function, so improving the intestinal immunity of fish is of great significance to aquaculture. Therefore, *L. helveticus* HML037, isolated from Inner Mongolia Koumiss, was evaluated as potential dietary supplement for *M. anguillicaudatus* fish by analyzing the effects on the intestinal health. The strain was administered in alginate-coated capsules containing 10^7^, 10^8^, and 10^9^ CFU for 8 weeks. The groups receiving *L. helveticus* HML037 had significantly improved growth rate, feed conversion ratio, condition factor, and survival rate. Intestinal digestive enzymes proteases, lipases, and amylases, that promote the absorption of nutrients, showed higher activities in the fish fed with *L. helveticus* HML037, mainly in the middle *L. helveticus* HML037 dose of 10^8^ CFU/g. Moreover, *L. helveticus* HML037 treatments significantly increased the activity antioxidant enzymes LZM, SOD, AKP, and CAT and decreased the MDA levels. After 8 weeks *TLR1*, *GH*, and *IGF-1* genes were significantly upregulated with the most significant effect the middle *L. helveticus* HML037 dose. The Shannon and Simpson diversity index in metagenome analysis of the intestinal microbiota indicated a higher community diversity in the *L. helveticus* HML037 groups with overrepresented *Pseudomonas*, *Flavobacterium*, *Actinomyces*, *Azoarcus*, and *Lactobacillus* genera [69].

## 7. *Lactococcus lactis*

*Lactococcus lactis* is one of the most common SLAB species used in the cheesemaking sector and naturally occurring in cheeses whose production implies the use of process temperatures favoring mesophilic microorganisms [23]. The effects reviewed for *Lactococcus lactis* by Illikoud et al. [4] include prevention of DSS-induced colitis in mice, with prevented release of NO and of inflammatory factors induced by lipopolysaccharides (LPS) in RAW264.7 cells, inhibition of *Salmonella* invasion into intestinal epithelial cells, production of an EPS found to increase macrophage phagocytosis, spleen and thymus indices and hemolytic complement (HC50) activity in cyclophosphamide-immunosuppressed mice. In clinical trials, milk fermented with *L. lactis* activated plasmacytoid dendritic cells, enhanced the ability to produce interferons, reduced symptoms such as sneezing and cumulative days of fatigue in athletes, and mitigated the severity of atopic dermatitis in children [4].

A recent review by Saleena et al. [70] summarized the in vivo immunological effects demonstrated for *L. lactis* strains of dairy origin and these included a reduction of IL-4 production in ovalbumin-sensitized mice, amelioration of recurrent colitis in mice, increased NK cell activities, concanavalin A-induced T cell proliferation, and serum levels of tumor necrosis factor (TNF)-α, IFN-γ, IL-2, IL-4, IL-10, and IL-12 in cyclophosphamide immunosuppressed mice, antihypertensive effect of the fermented milk in spontaneously hypertensive rats, stabilization of cancer cells in the colorectal epithelium, restored T cell population in small intestinal lamina propria in aged interleukin-18 deficient mice, and exerted protection against influenza virus infection. A clinical trial demonstrated that *L. lactis* ameliorated acute psychological stress in men.

### 7.1. Anti-Cancer Effects

Barcellos Jaskulski et al. [71] showed that a suspension of 10^8^ CFU/mL of *L. lactis* R7, isolated from ricotta and able to produce nisin, exerted anti-carcinogenic potential against colorectal epithelium cancer cells induced in Wistar rats with 1,2-dimethylhydrazine (DMH) when administered for 20 days. *L. lactis* R7 prevented the formation of cells with malignant atypia and of large infiltrate of lymphocytes in most animals, demonstrating an improvement of the immune response. Determination of hematic glutamic–oxalacetic transaminase (GOT) and glutamic–pyruvic transaminase (GPT) levels indicated that *L. lactis* R7 reduced hepatic cell damage caused by the oxidative effect of DMH.

### 7.2. Immunomodulation

The immunostimulating effects and associated mechanisms of *L. lactis* GCWB1176 isolated from mozzarella cheese were investigated in cyclophosphamide-induced (CTX-induced) immunosuppressed male ICR mice. *L. lactis* GCWB1176 was administered intragastrically at 10^7^ and 10^9^ CFU daily for 16 days. Mice were treated on days 7–9 by intraperitoneal injection of CTX. The body weight, spleen and thymus indices, and the cytokine TNF-α, IFN-γ, IL-2, IL-4, IL-10, and IL-12 levels in serum of the *L. lactis* GCWB1176-treated group increased dose-dependently. However, all doses of *L. lactis* GCWB1176 enhanced NK cell activity and lymphocyte proliferation, indicating that *L. lactis* GCWB1176 can alleviate CTX-induced immunosuppression [72].

### 7.3. Improvement of Stress Symtoms

*L. lactis* WHH2078 isolated from yogurt was administered in numbers of 10^9^ CFU/mL in mice with induced chronic unpredictable mild stress (CUMS) for 5 weeks and significantly ameliorated depressive and anxiety-like behaviors in the tail suspension test, forced swim test, sucrose preference test, and open field test. These effects were associated with a significantly reduced serum corticosterone level and restored levels of 5-hydroxytryptamine (5-HT, serotonin) and its precursor 5-hydroxytryptophan (5-HTP), and brain-derived neurotrophic factor. The gene for tryptophan hydroxylase Tph1 involved in synthesis of 5-HT, whose metabolism is associated with depression, was upregulated in the colon. CUMS was associated to colon dysbiosis. The high-throughput 16S rRNA gene sequencing of feces microbiota showed that *L. lactis* WHH2078 restored alpha diversity and the abundances of Firmicutes and Bacteroidetes, which is associated with the improvement of 5-HT metabolism [73].

### 7.4. Improvement of Depressive Behavior

The interaction between the intestinal microbiota, the gut, and the central nervous system (CNS), known as the brain–gut–microbiota axis, is increasingly studied for the possible applications in the treatment of neuropsychiatric disorders. Several preclinical studies have shown that probiotics may be used to modulate depressive-like behaviors [74].

*L. lactis* subsp. *cremoris* LL95, a LAB isolated from cheese was investigated in this respect by supplying daily 10^9^ CFU of it to female C57BL/6 mice for 28 days. On day 28, the *L. lactis* LL95 group showed an increased number of LAB CFU that was 1.4 × 10^9^ per 100 mg of feces vs. 6.0 × 10^8^ in the control. The weight of the hippocampus increased by 33% in female mice fed with *L. lactis* LL95 and a significant decrease in hippocampal ROS levels and an increase in the Ferric Reducing Antioxidant Power (FRAP) was observed in LL95-fed mice. Treatment with *L. lactis* LL95 resulted in a decrease in immobility time in the tail suspension test (TST), Forced Swim Test (FST), and immobile episodes and increase of latency to immobility with a reduction of depressive-like behavior in mice. The administration of LL95 did not affect locomotor activity in the open field test (OFT) or in the Elevated Plus Maze (EPM), suggesting anxiolytic effects [74].

## 8. *Latilactobacillus sakei*

### Infection Mitigation

It has been observed that the Fulani tribes of Cameroon are more resistant to malaria than other West Africa ethnic groups. The Fulani have a tradition of milk production, processing, and consumption, and this has been considered a possible explanation for resistance to malaria. Moreover, in Sudan, cow’s milk fermented over a long period and added to another product (*oka*) to obtain *Biruni*, is traditionally used in the treatment of malaria [75].

*Latilactobacillus sakei* UB27 isolated from traditionally fermented milk was administered to Balb/c mice infected with the chloroquine sensitive *Plasmodium berghei* ANKA. After 7 or 14 days, a gradual and significant dose and duration of treatment dependent reduction of the level of malaria parasitemia was observed in the *L. sakei* group. A level of 2.7 × 10^9^ CFU/mL determined 100% parasitemia suppression on day 20 and prolonged survival of treated mice [75].

## 9. *Limosilactobacillus* Species

### 9.1. Alleviation of Aging Damages

*Limosilactobacillus fermentum* is one of the most common cultivable and predominant microbes in fermented dairy products. The *L. fermentum* strain MBC2 was isolated from Mozzarella di Bufala Campana (MBC) traditional Italian PDO (Protected Designation of Origin) cheese. In this cheese, *L. fermentum* is one of the main microbiota components together with *L. delbrueckii* and *Leuconostoc lactis*. This strain was evaluated for effects on longevity using wild-type *C. elegans* strain N2 and CL2166 (dvIs19 [(pAF15)gst-4p::GFP::NLS] III), *pept-1* (lg601), and CF1038 *daf-16* (mu86) mutant ss living hosts [76].

*C. elegans* is a small soil nematode used as an animal model for probiotic evaluation because it is easy to treat, has a short lifespan, can be safely used in the laboratory, and propagates through self-fertilization. It shares two-thirds of the genes related to human diseases and is used in studies on longevity, immunity, neurodegenerative diseases, fat storage, DNA damage, and apoptosis. Its simple immunity systems facilitate studies on the immune signaling pathways [77].

Worms were propagated on peptone-free nematode growth medium (NGM) and fed with a normal diet constituting 10 mg of *E. coli* OP50 or with *L. rhamnosus* GG and *L. fermentum* separately. The wild type nematodes fed with *L. fermentum* MBC2 showed 50% viability at day 18, while for the controls fed with *L. rhamnosus* GG and *E. coli* OP50, 50% viability was recorded at day 11 and 14, respectively, thus indicating a significantly extended lifespan for the *L. fermentum* MBC2 group. This effect was not observed with *L. fermentum* MBC2 heat-killed at 65 °C for 90 min. The intestinal microbial count of *L. fermentum* MBC2 group at 5 days was 2-fold and 4-fold higher than that of *E. coli* OP50 and *L. rhamnosus* GG, respectively. Moreover, nematodes fed with *L. fermentum* MBC2 displayed a higher motility from 6 to 9 days and a higher pharyngeal pumping frequency, an indicator of food intake ability that declines with age, at 13 days. Intracellular lipofuscin, which is a marker of aging cellular damage and is determined by autofluorescence, was lower in the group fed with *L. fermentum* MBC2 [76].

Dysregulation of lipid metabolism induced by aging, determined by BODIPY staining, detected lower amounts of intestinal lipid droplets in *L. fermentum* MBC2 and *L. rhamnosus* GG-fed worms. Extended longevity was shown to correlate with enhanced resistance against oxidative damage. In transgenic *C. elegans* with genotype GST4::GFP, defective in GST-4, a glutathionyl S-transferase with highest activity against lipoperoxides, microscopy analysis showed that fluorescence intensity of *L. fermentum* MBC2-fed worms was notably lower than that of OP50- and LGG-fed animals, indicating that in *L. fermentum* MBC2-fed worms the production of reactive oxygen species (ROS) was effectively reduced compared to control worms at 1 and 13 days of adulthood. In the *C. elegans* PEPT-1 mutant, defective in one of the major regulators of fat content, *L. fermentum* MBC2 did not induce a pro-longevity effect, though ROS levels were reduced [76].

*L. fermentum* DR9, isolated from raw cow’s milk, was administered to male Sprague Dawley rats subjected with D-galactose induced aging. Rats receiving *L. fermentum* DR9 had higher Firmicutes/Bacteroidetes ratio, with genera *Lactobacillus* and *Blautia* significantly higher and *Prevotella*, *Bacteroides*, and Erysiperotrichaceae significantly lower than positive controls receiving only D-galactose, indicating the ability to restore the phylum composition during the aging process. Aged rats receiving *L. fermentum* DR9 showed a higher amount of fecal 5-oxoproline, a product of GSH metabolism, that showed an active antioxidant activity with possible positive effects on the organism [78].

### 9.2. Infection Mitigation

*L. reuteri* strain 2892 isolated from camel’s milk exerted protective effects against *H. pylori*-induced gastritis in the stomach tissues of in C57BL/6 mice. A pretreatment with this strain significantly downregulated the *H. pylori* virulence factor *cag*A gene expression, upregulated the tight junction molecules (zona occludens (ZO-1), claudin-4), and suppressed metalloproteinase (MMP)-2 and MMP-9. Moreover, *L. reuteri* 2892 reduced the serum concentrations of pro-inflammatory cytokines interleukin (IL)-6, IL-1β, and INF-γ and increased the anti-inflammatory cytokine, IL-10. In addition, it showed anti-oxidative stress activity by regulating the levels of SOD and MDA, suggesting that *L. reuteri* 2892 can attenuate *H. pylori*-induced gastritis [79].

## 10. *Pediococcus acidilactici*

### 10.1. Amelioration of Metabolic Syndrome

Diabetes mellitus is a life-threatening metabolic disorder that affects hundreds million adults globally and causes millions of deaths annually. Inhibition of the carbohydrates-hydrolyzing enzymes α-glucosidase and α-amylase is the most efficient intervention to reduce the postprandial glucose level in blood in diabetes treatment. Since most of the chemical compounds and protein extracts able to inhibit these enzymes are partially effective and have side effects, i.e., gastrointestinal symptoms such as bloating, diarrhea, and abdominal pain [80], Al-Emran et al. [81] examined the possibility of selecting and evaluating the in vivo potential of probiotics of dairy origin to inhibit α-glucosidase and α-amylase. Among 77 lactic acid bacterial isolates from 20 different milk samples, four isolates inhibited both enzymes. These were identified as *Pediococcus acidilactici* on the basis of the *phe*S gene and whole genome sequence. Analysis of genome sequences by PathogenFinder indicated a nonpathogenic probability value between 75.5% and 81.2% and the presence of antibiotic resistance (AR) genes in two isolates. All four isolates were used for an in vivo trial of the hypoglycemic effect in Swiss Webster male mice to which about 10^9^ CFU of the bacteria were administered daily by oral gavage for 6 weeks. Diabetes was induced after the third week with a single dose of Streptozotocin in all mice except the negative control (NC) group. A significant drop in blood glucose was observed in groups receiving three of the isolates compared with the positive control (PC) that did not receive any treatment [81].

In the lipid profile, a significant decrease of LDL was observed in all probiotic groups, even compared to the NC group, while HDL, TG, and total cholesterol decreased slightly. Body weight was significantly lower in all probiotic groups compared to all controls, as well as the one receiving metformin as standard diabetes treatment (PCT) [81].

### 10.2. Defense from Pollutants

A high heavy metal (HM)-resistant strain, *P. acidilactici* GR-1, isolated from yak milk, significantly alleviated chromate toxicity in mice. Therefore, it was tested in a clinical trial to investigate the potential of the strain to reduce toxic metal levels in workers of the metal mining and smelting of copper (Cu), Ni, and cobalt industry in Gansu province who had elevated levels of Cu and Ni in the urine [82]. The participants were randomly divided into a group receiving yogurt containing 10^10^ CFU *L. delbrueckii* subsp. *bulgaricus* and *S. thermophilus* and another receiving yogurt added with *P. acidilactici* GR-1 in the same number for 12 weeks. The exclusion criteria were the use of antibiotics within 1 month before the study, regular use of probiotic supplements, lactose intolerance, alcohol or drug abuse, and presence of comorbidities. The participants were stratified according to blood Cu and Ni levels in three sub-groups with low, medium, and high levels of heavy metals. The high-level and medium-level groups of participants who consumed probiotic yogurt showed significantly decreased blood Cu levels after 4 weeks and 8 weeks. Similarly, in the high- and medium-level sets, a significant decrease in blood Ni levels was observed earlier in the probiotic yogurt group than in the conventional yogurt group. In the low-level set, no significant decrease was noted in blood Cu or Ni levels. The fecal samples showed elevated Cu and Ni levels compared with the baseline values after 12 weeks of the intervention, indicating increased HM excretion with yogurt ingestion. With the elimination of HM from the body, oxidative stress (OS) and inflammation indicators in serum, including catalase (CAT) activity, anti-inflammatory IL-10, and IL-4 levels, and proinflammatory IL-6 and IL-1β levels, also improved after 12 weeks of probiotic yogurt intake. SCFAs acetate, propionate, and butyrate significantly increased in the feces of the probiotic yogurt group, possibly due to the enrichment of SCFA-producing bacteria. After 4 weeks of probiotic yogurt consumption, *P. acidilactici* GR-1 was significantly enriched in the fecal samples of the participants and remained significantly higher after the completion of the trial, indicating good adherence and colonization capacity in the intestine. Metagenomic shotgun sequencing revealed that the fecal microbiota the worker group that consumed probiotic yogurt was enriched in KEGG orthologs involved in OS response, including SOD, CAT, GPX, and glutathione reductase (NADPH), suggesting the induction extensive activation of HM-induced OS defense mechanisms. After the probiotic yogurt intervention, ascorbate, aldarate, taurine, and hypotaurine metabolism were enriched in the fecal metabolome, while GSH metabolism and pantothenate and CoA biosynthesis were enriched in the serum metabolome. Histidine, α-linolenic acid, and D-glutamine and D-glutamate metabolisms were also enriched. Each of the enriched antioxidation-related metabolites correlated positively with most of the enriched species in feces [82].

An animal experiment with Kunming mice administered with a broad-spectrum antibiotic that later received CuSO_4_ as heavy metal source at a sublethal concentration and the probiotic bacterial strain *P. acidilactici* GR-1 at a dose of 10^9^ CFU daily, it was observed that the remediating effect of *P. acidilactici* GR-1 was reduced, thus demonstrating that the integrity of indigenous gut microbiota was essential for the protective effects [82].

## 11. *Propionibacterium freudenreichii*

Dairy propionibacteria (PAB) grow in cheese after lactic acid bacteria (LAB) since they can use lactic acid as main carbon source to produce propionic acid, acetic acid, and carbon dioxide. PAB synthesize beneficial molecules including B_9_ and B_12_ vitamins and propionic acid that promotes gut health [4]. Among PAB, *Propionibacterium freudenreichii* and *Acidipropionibacterium acidipropionici*, formerly *P. acidipropionici*, are included in the updated list of microorganisms with qualified presumption of safety (QPS) status of the European Food Safety Authority (EFSA) (https://zenodo.org/record/6902983#.ZDgW_fZByUk, accessed on 13 April 2023) and the species *P. freudenreichii* is most frequently isolated from dairy products [83].

Illikoud et al. [4] reported that *P. freudenreichii* strains administered to mice in different studies protected from trinitrobenzenesulfonic acid (TNBS) and dextran sulfate sodium (DSS) induced colitis and colitis caused by the pathogen *Citrobacter rodentium* attenuating weight loss, oxidative stress, and histopathological scores. These effects could depend on increased immunoglobulin A (IgA) secretion in the small bowel, increased expression of IL-10, restored occludin gene expression, and reduced induction of TNFα, IFNγ, and IL-17. Similar effects on cytokine induction were observed in mucositis, a painful inflammation and ulceration of the mucous membranes, occurring as an adverse effect of chemotherapy and radiotherapy cancer treatments and induced in mice with the chemiotherapic 5-fluorouracile (5-FU). *P. freudenreichii* CIRM-BIA129, which is the type strain of the species ATCC 9614 of unknown but, most probably, dairy isolation source, when administered in cheese, showed a high immunomodulatory effect [4].

*P. freudenreichii* KCTC 1063 isolated from Swiss type cheese protected rats from DSS-induced colitis by stimulating the expression of MUC2 or mucin 2, a glycoprotein that forms an insoluble mucous barrier on the gut epithelium, in intestinal goblet cells. This effect is explained with the production of propionate, the most effective SCFAs in MUC2 production stimulation. In a clinical pilot trial involving ulcerative colitis patients, *P. freudenreichii* ET-3 improved the clinical activity index and the endoscopic index. Moreover, consumption of milk fermented by *P. freudenreichii* JS reduced the serum level of high-sensitivity C-reactive protein (hsCRP), a stable and easy to measure biochemical marker of inflammation [84], in a RCT in healthy adults [4].

### 11.1. Amelioration of Metabolic Syndrome

Although the alteration of the gut microbiota in obesity is not clearly defined, dysbiosis occurs in obesity and main species of the gastrointestinal microbiota and their beneficial metabolites, such as SCFAs, vitamin B_12_, and indole, are lost, and intestinal permeability and endotoxemia are increased, which induces inflammation and gluconeogenesis in the liver, decreases satiety in the brain, and increases TG incorporation in adipose tissues. In addition, increased gut permeability maintains low-grade chronic inflammation [85]. Among probiotics studied to improve obesity, *P. freudenreichii* was considered by An et al. [85] for its capacity to produce bifidogenic compounds that promote the growth of *Bifidobacterium* spp. in the gut, vitamin B_12_, propionic acid, and surface proteins with immunomodulatory properties. Therefore, they evaluated the effect of live *P. freudenreichii* MJ2 or hkMJ2 on the prevention of lipid accumulation in adipocytes and its antiobesity and antidiabetic activity in high-fat diet (HFD)-induced obese mice. Both forms of the probiotic decreased body weight gain and liver and epididymal white adipose tissue weights in high-fat diet (HFD)-induced obese mice by 31% and 22.8%, respectively. The food efficiency ratio (FER) of the MJ2 and hkMJ2 groups significantly decreased. The expression levels of genes and proteins related to adipogenesis and lipogenesis significantly decreased, while the expression level of genes related to lipolysis (HSL and ATGL) and fatty acid β-oxidation (*CPT-1α* and *ACOX1*) increased in both *P. freudenreichii* MJ2 and the hkMJ2-treated groups. In addition, blood glucose and fasting insulin levels decreased in both groups with ameliorated insulin resistance, thus showing that *P. freudenreichii* MJ2 can alleviate obesity and metabolic syndrome [85].

### 11.2. Alleviation of Aging Damages

*P. freudenreichii* KCTC 1063 was shown to extend the lifespan of *C. elegans* compared with *E. coli* OP50 standard diet by Kwon et al. [77]. The effect was observed in *daf*-*16*, *jnk-1*, *skn-1*, or *daf-7* loss-of-function mutants but not in *pmk-1*, *sek-1*, *mek-1*, *dbl-1*, *daf-12*, or *daf-2* mutants, which suggests potential roles for these genes in *P. freudenreichii*-induced longevity in *C. elegans*. The mean lifespan of *P. freudenreichii*-fed *C. elegans*, compared with that of *E. coli* OP50-fed worms, increased by approximately 13% and the *P. freudenreichii*-fed worms were significantly smaller than the *E. coli*-fed worms. Genes related to lifespan extension and immune response, including genes involved in the p38 MAPK and the TGF-β pathways, were significantly upregulated. Moreover, *P. freudenreichii* increased resistance against a human pathogen, *Salmonella* Typhimurium, through the activation of *skn-1*, which is involved in pathogen resistance in *C. elegans* [77].

### 11.3. Infection Mitigation

Propionibacteria were tested for pathogen exclusion potential. It was reported that in poults raised until 7 weeks of age, *P. freudenreichii* B3523 strain of dairy origin reduced the cecal colonization of *Salmonella* Heidelberg and this was also observed in finishing turkeys of 12 weeks of age supplemented with 10^12^ CFU in 18.9 l (5 gallons) water of *P. freudenreichii* B3523 and challenged with 10^8^ CFU/turkey *Salmonella* Heidelberg at 11 weeks. In these animals, cecal colonization of the pathogen was reduced by more than 2.0 Log CFU/g and its dissemination in the spleen decreased [86].

Subsequently, Nair et al. [87] administered this strain to turkeys by adding 10^10^ CFU of it to 18.9 l of the drinking water until 7 or 12 weeks of age. Then, challenge with *Salmonella* Heidelberg was carried out by administering it in numbers of 10^6^/10^8^ CFU to each bird at week 6 or 11 in two different experiments. Cecal microbiome analysis carried out by Illumina MiSeq showed that the relative abundance of Firmicutes in week 12 was significantly higher in the *P. freudenreichii* group. In the group administered with only *Salmonella* Heidelberg genera, *Streptococcus*, *Gordonibacter*, and *Turicibacter*, known to be associated with inflammatory responses in birds and mammals, showed higher relative abundance [87].

Genera with increased abundance in the *P. freudenreichii* group two days post infection in growing turkeys were *Subdoligranulum* and *Faecalibacterium*, while the *Streptococcus* genus abundance decreased. Seven days post infection, no differentially abundant genera were identified. In finishing turkeys, the abundance of *Lactobacillus* and *Ruminococcaceae* NK4A214 and UCG.010 increased two days post infection, while seven days post infection genera with increased abundance were *Lactococcus*, *Erysipelatoclostridium*, *Leuconostoc*, *Butyricicoccus*, indicating that *P. freudenreichii* supplementation increased the relative abundance of beneficial microbiota that could mitigate the carriage of *Salmonella* Heidelberg in turkeys [87].

### 11.4. Improvement of Bone Health

The effects of probiotics on bone health have been studied since an association was found between gut microbiota and osteo-immunology. Therefore, Yeom et al. [88] evaluated the effect of *P. freudenreichii* MJ2, a probiotic with anti-inflammatory properties isolated from raw milk, on osteoblast differentiation, mineralization, and related signaling pathway in an osteoporosis mouse model. Osteoporosis is a condition in which bone mass decreases and bone structure deteriorates. This condition occurs with higher frequency in postmenopausal women because of the decrease in estrogen levels. Estrogen and progesterone therapy has as side effect an increased risk of breast cancer. Therefore, less harmful treatments to delay osteoporosis progression are highly desired. The evaluation of the effect of *P. freudenreichii* administration on this pathological condition was prompted from the observation that this bacterium increased the mRNA levels of genes homologous to vertebrate BMP-5 and BMP-8, which function in the development of cartilage and bone, respectively, in *C. elegans* [88].

The animal models used were ovariectomized (OVX) rats to which live *P. freudenreichii* MJ2 and heat killed cells (hkMJ2) were administered. After 16 weeks of treatment, live/dead MJ2 administration did not show hepatotoxicity. Bone Mineral Density (BMD) of the femur in OVX rats treated with hkMJ2 increased significantly compared to the control, showing prevention of bone loss. This effect was attributed to heat stable surface proteins that were found to increase the levels of mRNAs for osteoblast differentiation-regulated genes and osteoblast mineralization in the human fetal osteoblast cell line hFOB 1.19 [88].

*P. freudenreichii* MJ2 strain was also evaluated for a possible effect on the improvement of rheumatoid arthritis (RA) symptoms. In this disease, osteoclast differentiation is crucial for bone absorption, and osteoclasts are involved in bone destruction [89].

*P. freudenreichii* MJ2 was found to decrease expression of genes and proteins related to the receptor activator of nuclear factor-κB ligand (RANKL) induced osteoclast differentiation from macrophages in the murine macrophage cell line, RAW 264.7. A high dose (10^8^ CFU) or a low dose (10^7^ CFU) CFU of live or heat killed *P. freudenreichii* MJ2 was administered daily for 3 weeks to male DBA/1J mice of eight weeks. Then, collagen-induced arthritis (CIA) was induced by injecting bovine type II collagen (CII) in the tail and the administration of *P. freudenreichii* MJ2 was continued until the end of the experiment. Live *P. freudenreichii* MJ2 treatment did not significantly affect the incidence of arthritis but hkMJ2 significantly decreased the arthritis score with reduced joint destruction. Bone surface/bone volume (BS/TV), BMD, trabecular thickness (Tb.Th), trabecular number (Tb.N), and bone volume/tissue volume (BV/TV) significantly increased in treated mice, while trabecular separation (Tb.sp) significantly decreased. The groups receiving *P. freudenreichii* MJ2 showed a lower degree of bone erosion, reduced leukocyte infiltration, and cartilage damage. The anti-inflammatory cytokine, IL-10, was significantly increased in the high dose *P. freudenreichii* MJ2 group, while the expression level of *IL-17*, which leads to the worsening of RA, was significantly decreased in all *P. freudenreichii* MJ2 groups. Inhibited osteoclast differentiation was attributed to a decreased expression of osteoclast differentiation-related genes and increased osteoprotegerin (*OPG*)/*RANKL* ratio, thus to the inhibition of the *NF-κB* signaling pathway. It was concluded that both live and dead *P. freudenreichii* MJ2 can prevent or improve RA in an animal model [89].

## 12. *Streptococcus thermophilus*

*Streptococcus thermophilus* is a SLAB species well adapted to fermented dairy products and is used as commercial or natural starter in yogurt and different cheese types, among which Grana type cheeses and Pasta Filata cheeses [15,16]. Illikoud et al. [4] summarized the effects of *S. thermophilus* strain treatment in mice with DSS or trinitrobenzene sulfonate (TNBS) induced colitis reporting that *S. thermophilus* strains prevented and decreased symptom intensity of DSS and TNBS induced colitis, including bodyweight loss, gastrointestinal bleeding, and bacterial translocation into the colonic tissue and to the liver, macroscopic and histologic damage, immune stimulation, and associated inflammation, levels of inducible nitric oxide synthase positive (iNOs^+^) cells in the large intestines. This effect was attributed to antioxidative activity, as inferred from the decreased content of lipid peroxides in the colon mucosa, decreased DSS induced pro-inflammatory IL-17 for a decrease in the percentage of Th17 lymphocytes in the lamina propria. For a yogurt isolate, these effects were also exerted by the isolated exopolysaccharides that restored the expression of tight junction proteins claudin-1, occludin, and E-canderin [4].

*S. thermophilus* strains mitigated 5-FU induced mucositis in mice by reducing diarrhea and weight and appetite loss, and restoring the intestinal architecture with maintenance of the epithelium structure in the small intestine and colon and reduced intestinal inflammation. It was suggested that the protective mechanisms exerted by *S. thermophilus* in intestinal inflammation may involve immunomodulation mediated by the production of folate, riboflavin, and exopolysaccharides [4].

In clinical trials, *S. thermophilus* oral intake improved serum collagen type II C-telopeptide and serum CRP in knee osteoarthritis and symptoms of atopic dermatitis [4].

### 12.1. Amelioration of Metabolic Syndrome

Heat-inactivated *S. thermophilus* MN-ZLW-002, a strain isolated from a Chinese dairy product, significantly reduced weight gain after three weeks administration to male C57BL/6 HFD fed mice. Fasting blood glucose levels were significantly reduced after four and eight weeks and total TGs were significantly reduced after 12 weeks. Serum IL-1b and chemokine ligand 3 (CCL3) markers of pro-inflammatory responses decreased. Suppression of pre-adipocytes differentiation through immunity was one of the suggested mechanisms of action of heat inactivated *S. thermophilus* MN-ZLW-002. A possible underlying mechanism was the regulation of the host immune system via cytokine release [90].

According to the WHO, cardiovascular diseases (CVDs) have been considered the main cause of death globally, with approximately ten million affected people every year [91]. Hypercholesterolemia is a risk factor for such diseases based on a three times higher risk of heart attack in hypercholesterolemic individuals. Nonpharmacological cholesterol reduction methods are available in addition to available medications, and they do not pose adverse effects. One such strategy is the use of probiotics to improve lipid metabolism [92]. It was even proposed that hypercholesterolemic people may consume probiotics and/or prebiotics as food supplements to an appropriate diet instead of cholesterol-lowering drugs [93]. *S. thermophilus* MH422542 isolated from an Egyptian dairy product was selected among dairy isolates on the basis of auto-aggregation and co-aggregation, adhesion ability to human intestinal cells and to Caco-2 cells, cholesterol assimilation, β-galactosidase activity, and bile salts hydrolysis. When administered to Albino rats in milk fermented by the strain for five weeks and in number of 10^8^ CFU/mL, determined lower TG and total lipids concentrations in blood. As for other LAB tested in the same study, the group receiving *S. thermophilus* at 10^8^ CFU/mL initial level exhibited a reduction of fecal *Staphylococcus* sp. and coliforms. The histological examination of the liver did not reveal any tissue or cell shape alteration [6].

### 12.2. Anti-Cancer Effects

It was observed that *S. thermophilus* can reduce the risk of colorectal cancer (CRC) in mice via the β-galactosidase activity which releases galactose that perturbs cancer development signaling pathways. Therefore, Li et al. [94] examined the immunomodulatory effects of *S. thermophilus* ATCC 19258, isolated from pasteurized milk, on the intestinal mucosa azoxymethane-induced colon cancer in male C57BL/6J-*Apc*^Min^/J mice, which harbor a mutation in the tumor suppressor gene *Apc*. Mice received 2 × 10^7^ CFU of *S. thermophilus* ATCC 19258 or *E. coli* strain MG1655 (negative control) or a nonsteroidal anti-inflammatory drug that protects against CRC (positive control) by oral gavage, and they were raised until 20 weeks. The *S. thermophilus*-gavaged mice had a reduction in tumor number and size. The tumor-suppressive effect of *S. thermophilus* was further validated in C57BL/6 mice injected with azoxymethane, followed by oral administration of *S. thermophilus* ATCC 19258. Notably, HTS analysis showed that *S. thermophilus* administration caused the enrichment of the probiotic *Bifidobacterium* and *Lactobacillus* genera, in particular the species *B. choerinum*, *B. pseudolongum*, *B. coryneforme*, *L. reuteri*, *L. animalis*, and *L. acidophilus*. The observed activities were dependent on β-galactosidase activity, demonstrated by the fact that a β-galactosidase knockout *S. thermophilus* ATCC 19258 strain was ineffective, while administration of galactose decreased small-intestine tumor number in *Apc*^min/+^ mice [94].

### 12.3. Infection Mitigation

The yogurt isolate *S. thermophilus* KLDS 3.1003 was able to exert antimicrobial activities for the production of bacteriocin-coding peptides. It is also endowed with intact genes for acid tolerance, salt-resistance, and antioxidant activities that favor its effectiveness against foodborne pathogens [95].

When administered orally to BALB/c mice for 14 days challenged with *E. coli* and *S. aureus*, it restored pathogen-disrupted blood parameters HDL, LDL, TP, TG, AST, ALT, and some minerals [95].

Sepsis is caused by severe infections and can lead to life-threatening organ disfunction. A balanced gut microbiota has a protective role during systemic inflammation; therefore, it was hypothesized that probiotic supplementation could be an aid in the treatment of sepsis [96]. Seven 14-week-old male BALB/c (H-2Dd) mice were administered different doses of LPS, a well-known mediator of sepsis even causing septic shock in uncontrolled infection, by intraperitoneal injection and then received about 3 × 10^8^ CFU of the yogurt isolate *S. thermophilus* 19 selected for its capacity to suppress the inflammatory response in vitro. All LPS-treated mice exhibited a reduction in food and water intake, but mice receiving *S. thermophilus* 19 showed no changes in body weight, possibly for a better recovery favored by alleviation of inflammation. The expression of TNF-α, IL-1β, and IL-6 was lower in all organs in the *S. thermophilus* 19 group and all histopathologic scores were decreased based on structure conservation and degree of inflammatory cell infiltration for liver, lung, small intestine, and kidney. High throughput sequencing of the 16S rRNA gene of the cecum fecal microbiota revealed no effect on species richness of *S. thermophilus* 19 but different abundances of the genera *Fusobacterium* and *Flavonifractor*, which could be involved in immunomodulation [96].

### 12.4. Alleviation of Aging Damage

Two strains of *S. thermophilus* isolated from fermented milk, T-1 and 510, were fed to four-day-old young adult *C. elegans* worms in alternative to *E. coli* OP50 normal diet. At day 11, *S. thermophilus* fed worms showed significantly increased survival rate and at day 14, lipofuscin lipid peroxidation product autofluorescence significantly decreased in the *S. thermophilus* groups. Moreover, locomotive capability indicator of muscle function was vigorous in the latter groups on day 18. As determined by qRT-PCR, feeding with *S. thermophilus* upregulated the expression of *daf*-16 encoding an orthologue of a transcription factor that regulates longevity under the conditions of oxidative stress and caloric restriction. In fact, the expression of several antioxidant genes also increased. The results of qRT-PCR indicated activation of the DAF-7/TGF-β pathway as mechanism underlying the enhanced expression of *daf*-16, suggesting possible analogous effects of these universal pathways in other hosts [97].

### 12.5. Defense from Pollutants

A study investigated the effect of administration of *S. thermophilus* LM1012, isolated from a dairy product, on liver damage deriving from exposure to diesel exhaust particulate matter (DEPM). *S. thermophilus* LM1012 was administered in 10^6^ CFU/g to male 5-week old ICR mice daily for 14 days. Mice were then exposed to DEPM. Based on AST, ALT, and LDH levels in the blood, *S. thermophilus* LM1012 decreased liver injury induced by DEPM in mice [98].

## 13. Mixed Cultures

### 13.1. Amelioration of Intestinal Health

A mixed culture (YC) of *S. thermophilus* MK-10 and *L. bulgaricus* 151 starters with synergistic effects during yogurt fermentation was administered for 8 days in numbers of 5 × 10^9^ CFU to female specific-pathogen-free BALB/c mice after induction of colitis with DSS. The YC group showed attenuated severity of colitis, not decreased colon length and ameliorated histological signs. Myeloperoxidase (MPO) activity, an indicator of neutrophil infiltration into inflamed tissue and marker of acute inflammation, significantly decreased. YC lowered valeric acid and isovaleric acid and total putrefactive SCFA but did not show differences in the amounts of acetic, propionic, and lactic acids. Cytotoxic CD3^+^CD8^+^ T cells and CD3^+^CD4^+^CD25^+^FoxP3^+^ Tregs were downregulated in Peyer’s patches and CD3^+^CD4^+^CD25^+^ T cells increased in spleen and CD3^+^CD4^+^CD25^+^FoxP3^+^ cells in peripheral blood mononuclear cells. IgA antibody-forming cells were downregulated in mesenteric lymph nodes and enhanced in the spleen. The authors concluded that the strains possess the ability to modulate the intestinal mucosal and systemic immune system toward both IgA production and induction of Tregs cells by shifting Th1/Th2 balance [99].

### 13.2. Infection Alleviation

*L. delbrueckii* subsp. *bulgaricus* KLDS 1.0207 and *S. thermophilus* KLDS 3.1003 from Mongolian indigenous dairy product and traditional yogurt significantly suppressed the pathogenicity of *E. coli* ATCC 25922 and *S. aureus* ATCC 25923 female BALB/c mice. Standard hematological parameters of immunity showed that single cultures and co-cultures of the two strains could potentially improve immunity levels in humans [100]. When tested alone, *L. delbrueckii* subsp. *bulgaricus* KLDS 1.0207, at a 10^8^ CFU daily dose, determined a lower weight loss for the groups receiving both pathogens, restored the serum AST, total protein and albumin levels in the *E. coli* group, and brought AST, ALT, and albumin to normal levels in the *S. aureus* group [101].

Pretreatment with commercial dairy protective cultures reduced mortality caused by *L. monocytogenes* Scott A in *C. elegans*. *L. monocytogenes* in L4/young adult nematodes infected for 24 h reduced the 50% (TD_50_) survival from about 10 days to about 7 days. Pretreatment by plate feeding of *C. elegans* with *L. lactis* DVS BS-10 resulted in higher times of 50% survival. TD_50_ values for *L. plantarum* Holdbac *Listeria* and *L. plantarum* Lyofast were similar to those of the negative control fed with *E. coli* OP50. Pretreatment of *C. elegans* with different protective cultures did not affect the colonization levels of *L. monocytogenes*, suggesting that the reduced mortality should be attributed to the enhancement of the immune response [102].

## 14. *Enterococci* as Controversial Probiotics

Enterococci are very commonly found as a natural component of the cheese microbiota [8] and a few *Enterococcus* species were considered for use as probiotics, namely, *E. faecalis*, *E. faecium*, *E. lactis*, and more recently *E. hirae* and *E. durans*. Some *Enterococcus* strains are currently marketed to alleviate the symptoms of irritable bowel syndrome and recurrent chronic sinusitis or bronchitis. In particular, *E. faecalis* Symbioflor 1, isolated from the stool of a healthy human adult, has been used as a probiotic for more than 50 years without any reported adverse effect. It has also been shown to be nontoxic in *C. elegans*. Based on several toxicological studies, that are not publicly available, long time of safe use, and on the genome sequence [103], this strain can be administered to humans. However, some enterococci can behave as opportunistic pathogens and are a prevalent cause of nosocomial infections such as bacteremia, endocarditis, urinary tract infections, and other infections [104]. Virulence genes that can be found in *E. faecalis* encode an aggregation substance (*asa1*), a gelatinase (*gelE*), a collagen adhesin (*ace*), an enterococcal surface protein (*esp*), and a cytolysin (*cylA*) and are sporadically detected in dairy isolates. For the possible presence of these virulence genes, *Enterococcus* species are not included in the QPS list [EFSA], so that in potential probiotic strains the absence of transferable AR genes and virulence determinants must be ascertained before use in the pharmaceutical or food industry. On the other hand, horizontal transfer of AR genes among different strains was demonstrated in vivo in volunteers, such that AR genes represent a risk for the spread of resistance genes to indigenous enterococci, which can be responsible for infections in immunocompromised patients [105].

Among isolates from Tunisian dairy products, one of the most promising strains in in vitro preliminary testing, *E. faecalis* OB14, led to high mortality of *Galleria Mellonella* larvae in the virulence test. Another strain, *E. faecalis* OB15, devoid of cytolysin genes, was not toxic for the larvae. MALDI TOF-MS and WGS analyses showed that *E. faecalis* OB15 was closely related to the *E. faecalis* Symbioflor 1 [104]. This indicates the opportunity to test the probiotic potentialities of individual strains in in vivo models.

In a study aimed to distinguish potentially pathogenic from non-pathogenic enterococci, it was found that 30 isolates from different dairy products carried on average four AR genes and all carried at least one AR gene. All isolates were classified as potential human pathogens by PathogenFinder analysis. However, *E. faecium* isolates, that were the most prevalent, showed the absence of adhesins *sgr*A, *ecb*A, and *scm* virulence factors present in human pathogenic strains [106].

### 14.1. Immunomodulation

Darwish et al. [6] found that in Albino rats fed with fermented milk prepared with *E. faecium* MH422543, preliminarily evaluated for absence of virulence factors, 10^8^ CFU/mL of the strain administered for five weeks determined increased G3 lymphocytes in blood, indicating an immune response. The *E. faecium* group had higher concentrations of TG and total lipids than other treatments compared but a remarkable suppression of LDL and 88% reduction of atherogenic indices.

### 14.2. Infection Mitigation

Despite carrying some virulence factors and antibiotic resistance genes, *E. faecalis* UGRA10 isolated from a Spanish sheep’s cheese was administered with a final concentration in water of 10^9^ CFU/mL for 30 days to rainbow trout before challenging by intraperitoneal injection of 5 × 10^8^ CFU/fish of the fish pathogen *L. garvieae* CECT 5274 [107]. The cumulative survival rate of the treated trout was 50% compared with 0% for the control fish. *E. faecalis* UGRA10 produces enterocin AS-48, a cationic circular bacteriocin with broad bactericidal activity against most Gram-positive and some Gram-negative bacteria. Trout inoculated with the pathogen and treated by regular dipping in AS-48 baths had a survival rate of 60% after 20 days compared with that of untreated fish (0%). Therefore, the isolated bacteriocin, which was shown not to be toxic in the trout, can substitute use of *E. faecalis* UGRA10 as alternative to antibiotics to control *L. garvieae* infections in aquaculture [106].

## 15. Overview on In Vivo Beneficial Effects of Dairy Bacteria

**Figure 1:**
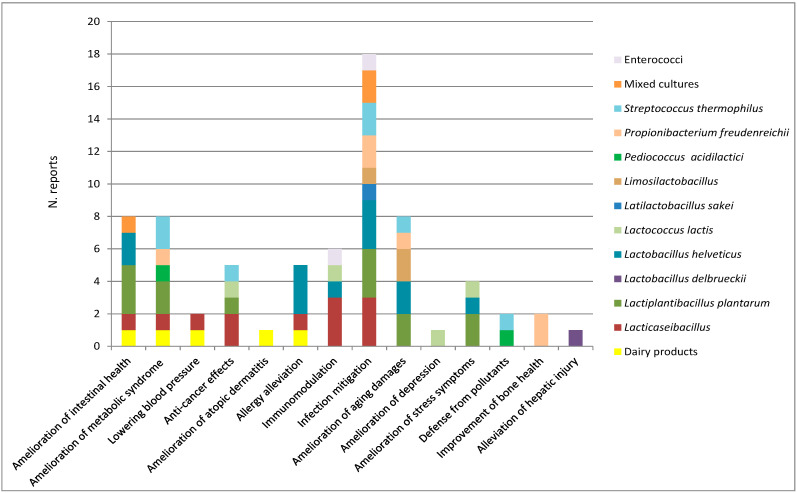
Number of reports summarized in this review for each health issue and dairy microorganisms involved.

The number of reports summarized in this review separated by topic and microorganisms involved are shown in Figure 1.

Figure 1 is a visual summary of the in vivo studies commented on in this review and shows that most of these regarded infection mitigation, for which nearly all the bacterial groups considered were tested. Among these, the *Lacticaseibacillus* genus, *L. helveticus*, and *L. plantarum* were proven to be beneficial in a higher number of disease conditions. The species *P. acidilactici* and *S. thermophilus* were the only ones tested in the detoxification of harmful substances, and the encouraging results obtained indicated that dairy bacterial strains should be further tested against the detrimental effects of exposure to pollutants. Strains of *P. freudenreichii* were proven to be beneficial in bone health and their application in this field should move forward to clinical trials. Importantly, *L. delbrueckii* subsp. *bulgaricus*, a species used for the production of yogurt, the most widespread industrial fermented milk, was proven to alleviate hepatic injury. Therefore, the intra-species selection of strains of this SLAB with the same activity and their use in yogurt production could be beneficial to a large number of people.

Studies on changes of the intestinal microbiota induced by dairy bacteria should be re-interpreted based on novel findings regarding particular bacterial groups, as in the case of *Desufovibrio* genus. This was found to increase after *L. plantarum* DR7 treatment by Liu et al. [53], but it was recently reported that some strains potentially worsen Parkinson’s disease based on evidence in a *C. elegans* model [108].

## 16. Conclusions

This review on the in vivo health promoting effects of microorganisms of dairy origin highlights that multiple advantages could derive from the evaluation of the characteristics and selection of strains with enhanced capacity to ameliorate different disease conditions. Since fermented dairy products supply high numbers of bacterial species comprising strains with probiotic properties, more studies on the in vivo effects of the microorganisms present could make available probiotics well adapted to dairy foods to increase their functional role.

## Data Availability

Not applicable.
